# Comparative assessment of large language models in diabetic foot infection management: alignment with IWGDF/IDSA guidelines

**DOI:** 10.3389/fendo.2026.1667159

**Published:** 2026-02-24

**Authors:** Hongxia Wu, Jiayi Deng, Xu Qiu, Li Xu, Lumeng Lu, Mingna Fan, Danni Yu, Chuanbo Liu, Zhaohuan Chen, Kai Wang, Yuyan Wang, Haifang Zhou, Liyang Chang, Hanbin Wang

**Affiliations:** 1 Emergency Department, Hangzhou Traditional Chinese Medicine Hospital Affiliated to Zhejiang Chinese Medical University, Hangzhou, China; 2 Department of Pain, The Affiliated Hangzhou First People’s Hospital, Westlake University School of Medicine, Hangzhou, China; 3 The Fourth Clinical School of Medicine, Zhejiang Chinese Medical University, Hangzhou, China; 4 Nursing Department, Hangzhou Traditional Chinese Medicine Hospital Affiliated to Zhejiang Chinese Medical University, Hangzhou, China; 5 Department of Plastic and Cosmetic Surgery, The Affiliated Hangzhou First People’s Hospital, Westlake University School of Medicine, Hangzhou, China; 6 Department of Vascular and Hernia Surgery, The First People’s Hospital of Hangzhou Lining District, Hangzhou, China

**Keywords:** adherence, artificial intelligence, diabetic foot infection, guideline, large language models

## Abstract

**Objective:**

To assess the clinical utility of artificial intelligence (AI) models (ChatGPT-4o, DeepSeek-R1, Grok-3 and Claude-3.7) in aligning with international guidelines for diabetic foot infection (DFI) management.

**Background:**

AI systems have demonstrated their potential application value in numerous fields. However, the specific effects of these technologies in the medical and health sector still require in-depth exploration. DFI is a relatively common and serious complication among diabetic patients, and the accurate transmission of relevant information is of great significance. Therefore, it is particularly important to evaluate whether artificial intelligence can serve as an effective clinical auxiliary tool.

**Methods:**

Responses from ChatGPT-4o, DeepSeek-R1, Grok-3 and Claude-3.7 were evaluated against DFI guidelines using four clinical dimensions (Accuracy, Overconclusiveness, Supplementary Value, and Completeness) using a 5-point Likert scale, and assessed for readability using Flesch Reading Ease (FRE) and Flesch–Kincaid Grade Level (FKGL). Statistical analyses included ANOVA and *post hoc* comparisons.

**Results:**

No significant differences were found across models for Accuracy and Overconclusiveness (*p* > 0.05). However, Supplementary Value differed significantly (*p* < 0.001), the performance of Grok-3 is superior to that of ChatGPT-4o (*p* < 0.0001), DeepSeek-R1 (*p*=0.003), and Claude-3.7 (*p* < 0.0001). Meanwhile, there are significant differences in terms of Completeness (*p*=0.005), Grok-3 outperforms ChatGPT-4o (*p*=0.016)and Claude-3.7 (*p*=0.010) significantly.Readability also varied: DeepSeek-R1 responses were more complex than ChatGPT-4o (*p* = 0.046).

**Conclusion:**

All models perform comparably in terms of accuracy and in avoiding over-conclusions. Grok-3 outperformed the other models in the dimensions of complementarity and completeness. DeepSeek-R1 generated the most complex text. These findings validate the feasibility of AI in the standardized management of DFI, but the models still need to be further verified through clinical trials to determine their value in the real-world decision-making process.

## Introduction

Diabetic foot infection (DFI) has become a major challenge in the global medical field (1, 2), affecting approximately 19% to 34% of diabetic patients. Within five years of diagnosis, 17% of patients may progress to lower limb amputation (3). Despite the complexity of the treatment process, the guidelines jointly released by the International Working Group on the Diabetic Foot (IWGDF) and the Infectious Diseases Society of America (IDSA) in 2023 (4) provide a gold standard approach for assessing the severity of infection (IDSA/IWGDF 2023 classification), selecting antibiotics based on local antimicrobial susceptibility patterns, and establishing surgical referral criteria. With the rapid development of artificial intelligence technology, large language models (LLMs) have gradually become important tools for patients, medical students, and clinicians to obtain relevant information. However, due to the fact that these models have not been strictly validated to ensure the medical accuracy of their output information, concerns have been raised about their reliability in providing medical advice (5, 6).

The advent of generative artificial intelligence (AI) models, exemplified by systems like ChatGPT, represents a significant milestone in the evolution of healthcare technologies. These advanced AI systems leverage deep learning architectures, particularly transformer-based neural networks, to process and generate human-like text (7, 8). A key feature of these models is their ability to employ a multi-step “Chain-of-Thought” (CoT) reasoning based on probabilistic inference, which enables them to excel in structured reasoning tasks and generate logically coherent suggestions to support clinical decision-making (9, 10). By breaking down complex medical queries into sequential reasoning steps, generative AI can mimic the cognitive processes of healthcare professionals, offering potential benefits in diagnostic accuracy, treatment planning, and patient education. However, the rapid integration of AI technologies into healthcare also raises critical concerns regarding their alignment with evidence-based clinical guidelines. LLMs such as ChatGPT-4o, DeepSeek-R1, Grok-3 and Claude-3.7 while increasingly accessible to both patients and clinicians, face unresolved questions about their reliability in delivering medically accurate recommendations without rigorous validation (11, 12). Although prior studies have evaluated LLMs in specialties such as orthopedic pathology and stroke severity (13, 14), the existing review and bibliometric studies reflect that LLMs applications in DFI remain unexplored (15, 16). DFI is a complex condition requiring integrated management of infection severity grading, antimicrobial stewardship, and surgical timing decisions.

Our study conducts a systematic evaluation of LLMs adherence to the 2023 IWGDF/IDSA guidelines, which are widely regarded as the benchmark for managing DFI. By analyzing model-generated responses to standardized clinical scenarios spanning infection classification, antibiotic selection, and surgical referral criteria, we quantify fidelity to evidence-based protocols and identify critical discrepancies in clinical reasoning. These findings will facilitate the implementation of LLMs. They ensure patient safety by following the recommendations of the guidelines, and reducing the workload of doctors through automated and context-based decision support, thereby promoting the safe integration of artificial intelligence into the diabetes foot care pathway.

## Methods

This study utilized a suite of publicly accessible LLMs, specifically ChatGPT-4o, DeepSeek-R1 with Deep Think functionality, Grok-3, and Claude-3.7, to conduct its analyses. Given the non-human subject nature of the research, institutional review board (IRB) approval was not required, in accordance with ethical research guidelines.

To ensure the impartiality and reliability of the responses generated by these models, a rigorous methodology was employed. Each question was submitted independently to the respective versions of ChatGPT-4o, DeepSeek-R1, Grok-3, and Claude-3.7 without any prior prompting or contextual carryover from previous interactions. To further enhance the integrity of the results, a fresh chat session was initiated for every individual question, thereby minimizing any potential residual influence from prior queries. In the application of these AI models, the phenomenon of the priming effect is a critical consideration. The priming effect refers to the subtle influence that prior input data or contextual cues may exert on a model’s output, potentially leading to biased tendencies, defined response patterns, or skewed interpretations. To mitigate the risk of such priming effects, strict temporal separation was maintained between interactions with different model versions. This was achieved by establishing a new session window for each question, ensuring that no residual context from previous queries could inadvertently shape the responses. This methodological rigor was implemented to uphold the objectivity and reproducibility of the findings. Furthermore, models including ChatGPT-4o, DeepSeek-R1, Grok-3, and Claude-3.7 are openly available. Their documented significance in contemporary medical literature indicates a strong potential for enhancing clinical workflows (17–20).

A 5-point Likert scale was used to assess the accuracy and completeness of the four models responses: Accuracy, Overconclusiveness, Supplementary Value and Completeness (13, 21).

Accuracy:

1. Completely incorrect
2. More incorrect than correct [> 75% incorrect]
3. Approximately equal correct and incorrect
4. More correct than incorrect [> 75% correct]
5. Completely correct

Overconclusiveness:

1. Non-overconclusive [0% conflicting]
2. Minimally overconclusive [<25% conflicting]
3. Partially overconclusive [50% conflicting]
4. Mostly overconclusive [>75% conflicting]
5. Fully overconclusive [100% conflicting]

Supplementary Value:

1. No supplementary value [0% added]
2. Low supplementary value [25% added]
3. Moderate supplementary value [50% added]
4. High supplementary value [>75% added]
5. Exceptional supplementary value [100% novel]

Completeness:

1. Very incomplete [0–25%]
2. Incomplete [25–50%]
3. Moderate [50–75%]
4. Complete [> 75%]
5. Very complete [100%]

Additionally, to assess the readability of each model’s responses, we calculated the Flesch Reading Ease (FRE) and Flesch–Kincaid Grade Level (FKGL) scores for each model’s responses (21). A higher FRE score indicates easier readability, while a lower FKGL suggests the text is suitable for readers at a lower grade level.

The evaluation of the responses of the four models, namely ChatGPT-4o, DeepSeek-R1, Grok-3 and Claude-3.7, was carried out by 3 independent reviewers to ensure the reliability of the scoring process. In cases of disagreement, a fourth author was consulted for resolution. SPSS 26.0 was used for statistical analysis. One-way analysis of variance (ANOVA) was applied to compare performance differences across models under identical dimensions. Fleiss’ kappa, serving as a generalization of this statistic, was used in SPSS to evaluate the consistency among the three raters for the ChatGPT-4o, DeepSeek-R1, Grok-3 and Claude-3.7 response qualities. *p* < 0.05 was considered to have statistical significance.

## Results

In this study, we evaluated the performance of four LLMs—ChatGPT-4o, DeepSeek-R1, Grok-3 and Claude-3.7. There were a total of 7 clinical scenarios that were included in the consensus practice guidelines on DFI. The outputs were evaluated using a 5-point Likert scale across four dimensions: Accuracy, Overconclusiveness, Supplementary Value, and Completeness. This approach allowed for a comprehensive comparison of each model’s alignment with established clinical guidelines. The responses generated by all evaluated models were systematically documented in **Supplementary Table 1**.

**Figure 1:**
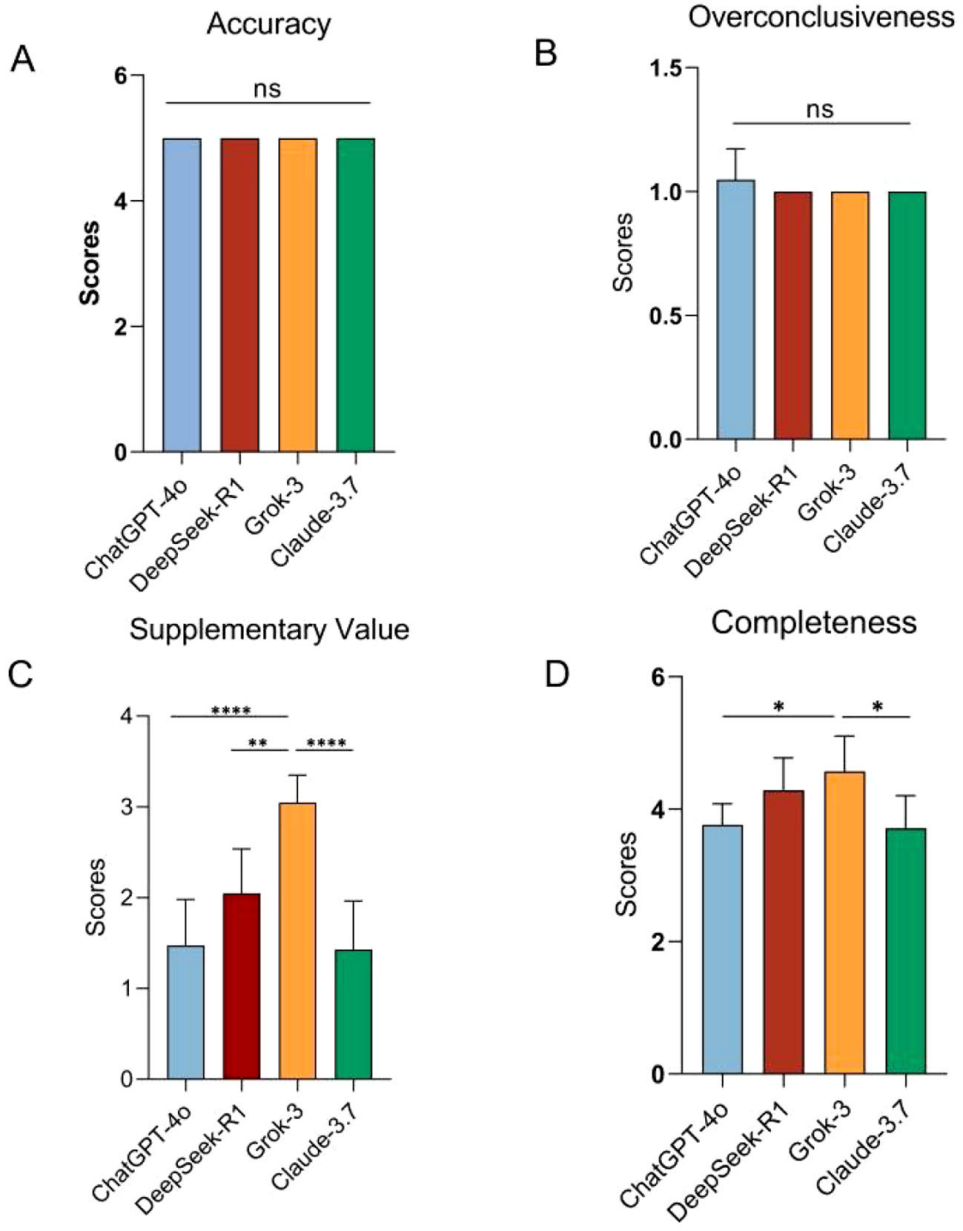
Comparison among models. **(A)** Comparison of accuracy value scores across models. **(B)** Comparison of overconclusiveness value scores across models. **(C)** Comparison of Supplementary Value Scores Across Models. **(D)** Comparison of Completeness Value Scores Across Models. *p < 0.05,**p < 0.01,****p < 0.0001, ns, not significant.

Using a 5-point Likert scale, no significant differences were observed among the four models in terms of Accuracy (*p* = 1), Overconclusiveness(*p* = 0.410). Significant differences in Supplementary Value were observed among the ChatGPT-4o, DeepSeek-R1, Grok-3 and Claude-3.7 (one-way ANOVA: F(3, 24) = 17.67, *p* < 0.001, R² = 0.688). *Post hoc* Tukey’s HSD tests revealed Grok-3 had significantly higher supplementarity than ChatGPT-4o (*p* < 0.0001), DeepSeek-R1 (*p* = 0.003), Claude-3.7 (*p* < 0.0001). No significant differences were detected among ChatGPT-4o, DeepSeek-R1 and Claude-3.7 (all *p* > 0.05). Despite the ordinal pattern Grok-3 (3.049 ± 0.300) > DeepSeek-R1 (2.047 ± 0.489) > ChatGPT-4o (1.476 ± 0.504) > Claude-3.7 (1.429 ± 0.535), statistical significance was confined to the contrasts between Grok-3 and the remaining groups. Among the four AI models, namely ChatGPT-4o, DeepSeek-R1, Grok-3 and Claude-3.7, there are significant differences in terms of completeness (one-way ANOVA: F(3, 24) = 5.622, *p* = 0.005, R² = 0.413). *Post hoc* Tukey’s HSD tests revealed Grok-3 had significantly higher supplementarity than ChatGPT-4o (*p* = 0.016), Claude-3.7 (*p* = 0.010). No other pairwise comparisons reached statistical significance (adjusted *p* > 0.05 for all other model combinations) (**Figure 1**).

**Figure 2:**
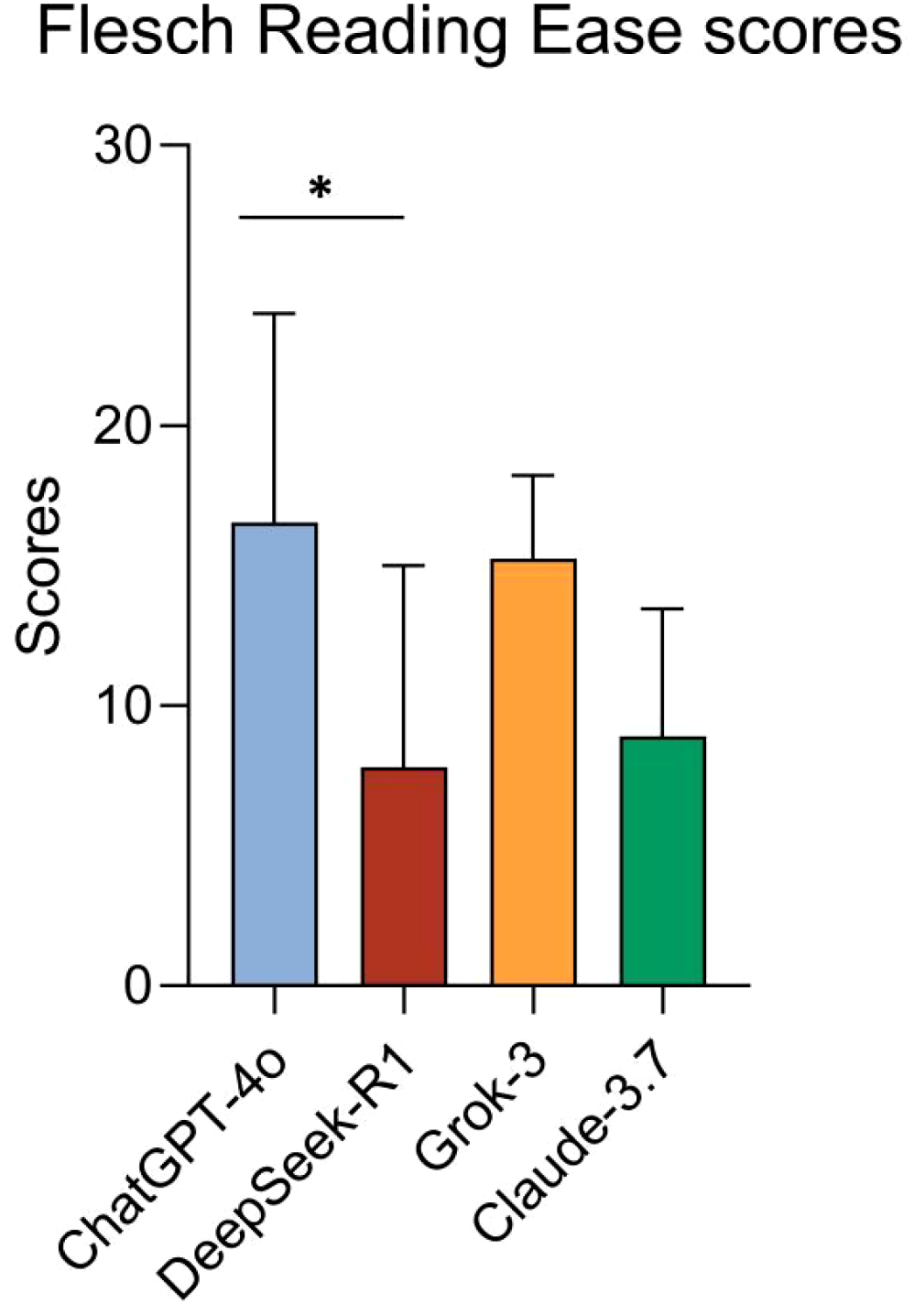
Comparison of FRE scores across models. *p < 0.05.

Significant differences in FRE scores were observed among the four models. One-way ANOVA showed notable group differences (F(3, 24) = 3.993, *p* = 0.019, R² = 0.3329). *Post hoc* tests revealed DeepSeek-R1 had significantly lower readability than ChatGPT-4o (adjusted *p* = 0.046, 95% CI: 0.1296 to 17.37). No other pairwise comparisons reached statistical significance (adjusted *p* > 0.05 for all other model-strategy combinations) (**Figure 2**).

**Figure 3:**
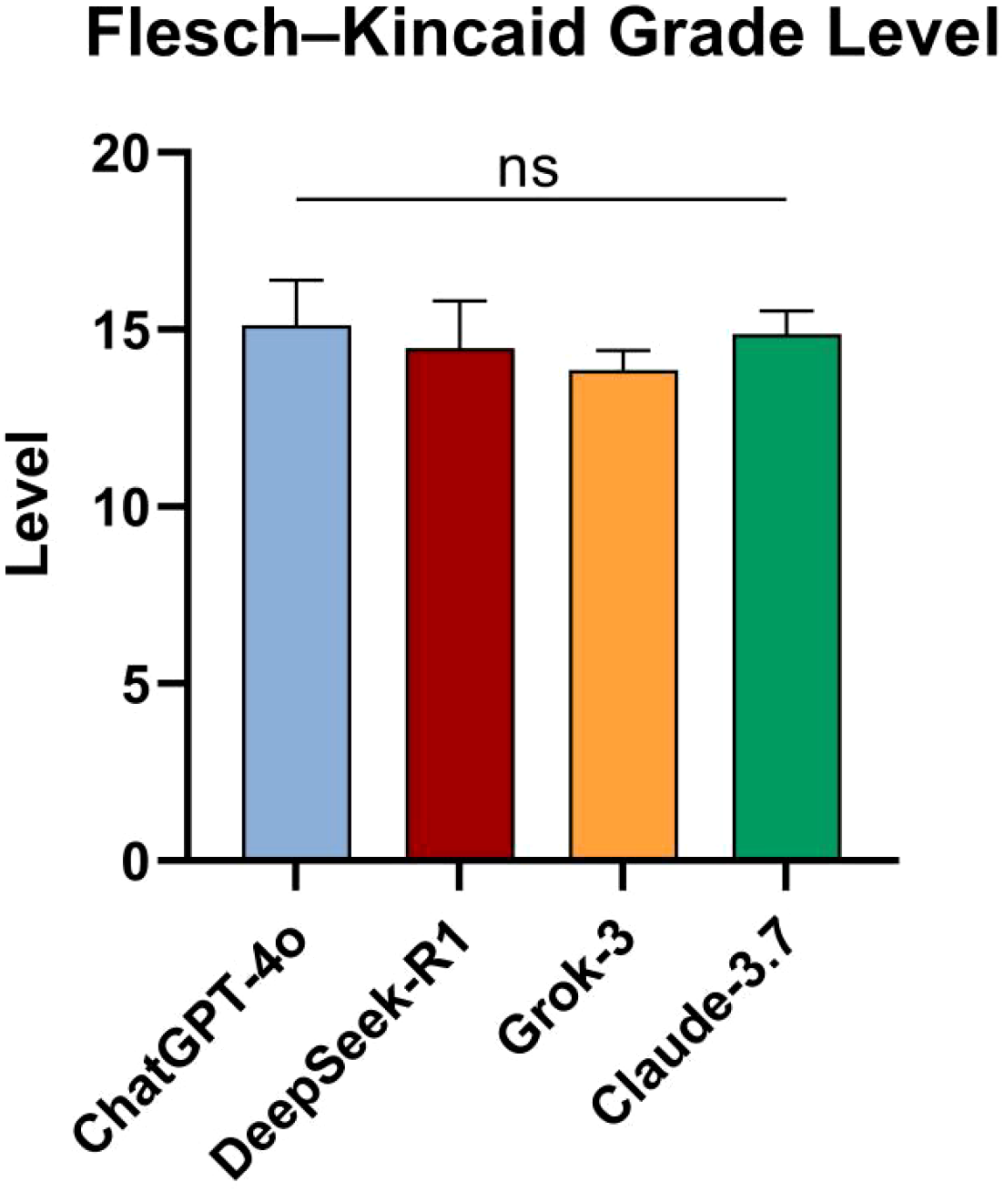
Comparison of FKGL across models. ns, not significant.

No significant differences were observed among the four models in terms of FKGL (*p* = 0.128) (**Figure 3**).

The Kappa consistency analysis revealed that the consistency among the evaluators reached an almost perfect level (κ = 0.890, *p* = 0.001). According to the Landis & Koch criteria, this result was highly statistically significant, indicating that the consistency among the evaluators was not caused by random factors.

## Discussion

DFI is one of the most serious complications of diabetes. Approximately 6.3% of diabetic patients worldwide are at risk of foot ulcers, and half of them will subsequently develop an infection. Such infections account for 85% of all diabetic-related amputations each year (22–24). Its pathogenesis is driven by neuropathy, ischemia, and immune disorders, leading to difficult-to-heal multi-microbial infections (25, 26). Dickson et al. (27) analyzed 2.4 million emergency cases involving DFI in the United States from 2012 to 2021. The results showed that patients with diabetic foot infection had a threefold higher chance of hospitalization compared to those without DFI (OR = 3.002, *p* < 0.001), and their hospital stay was prolonged by 55%, significantly increasing the social and economic burden (1). As various forms of AI are integrated into medical settings, the reliability of AI-generated content needs to be fully assessed. This study evaluates the concordance of ChatGPT-4o, DeepSeek-R1, Grok-3 and Claude-3.7 with the 2023 international consensus guidelines for DFI. The findings highlight both the strengths and limitations of AI models in supporting clinical decision-making, emphasizing the need for careful integration and human oversight to ensure optimal patient care.

Across the dimensions of Accuracy and Overconclusiveness, no statistically significant differences were observed among the ChatGPT-4o, DeepSeek-R1, Grok-3 and Claude-3.7. This aligns with recent advancements in AI models’ capacity to process structured clinical guidelines and generate evidence-based recommendations (28). For instance, ChatGPT-4o leverages its robust training framework to maintain guideline compliance despite architectural constraints, DeepSeek-R1’s open-source architecture allows for rapid adaptation to domain-specific protocols, Grok-3 minimizes speculative outputs through its deep search and structured validation mechanisms, Claude-3.7 allows it to balance efficiency with deep, stepwise analysis in complex clinical scenarios while strictly avoiding speculative outputs (29, 30). Notably, the absence of overconclusiveness in four models suggests robust alignment with guideline principles. This restraint helps avoid speculative recommendations in areas where evidence is insufficient, thereby providing a critical safeguard against iatrogenic risks in DFI management.

In contrast, the models demonstrated statistically significant differences in terms of Supplementary Value, with Grok-3 generally outperforming ChatGPT-4o, DeepSeek-R1 and Claude-3.7. For instance, when addressing pathogen identification in diabetic foot infections to guide antibiotic therapy, Grok introduced novel testing methods like polymerase chain reaction (PCR) and next-generation sequencing (NGS), along with operational details such as debridement techniques. This finding indicates that model architecture substantially influence the depth, contextuality, and educational value of responses (20). At the same time, these supplementary information can further enhance the quality of clinical decision-making by providing broader background knowledge or the latest research findings that have not yet been included in the guidelines. For instance, AI can deeply analyze the pathophysiological mechanisms of DFI and its related risk factors, which is of great reference value for patient education and for clinicians who wish to acquire the latest knowledge. However, such supplementary information needs to undergo strict evaluation and validation. As pointed out by Giuffre et al., the accuracy and relevance of these details may vary, and there is a risk of deviating from the guideline-oriented nursing process due to information overload (11, 31).

Although the models demonstrated generally high accuracy, their outputs exhibited deficiencies in completeness. These omissions frequently involved critical details such as pathogenesis mechanisms, including biofilm dynamics in chronic ulcers, and comprehensive multimodal treatment protocols that combine offloading with antibiotic stewardship. This limitation may originate from the reliance of LLMs on textual data rather than integrated multimodal inputs such as imaging or biomarkers, which are essential for holistic DFI care. For instance, while the IWGDF guidelines emphasize the importance of the Wound, Ischemia, and foot Infection (WIfI) classification system for ischemia assessment, AI models often fail to explicitly contextualize perfusion scores within broader treatment algorithms. A similar limitation has been observed in studies on AI-driven diabetic retinopathy tools (32). It is worth noting that Grok-3 performed better in terms of completeness than ChatGPT-4o and Claude-3.7, which may be related to its Chain of Thought reasoning mechanism and DeepSearch capabilities, enabling stepwise decomposition of complex clinical scenarios and real-time integration of multimodal evidence.

The Fleiss’ Kappa value of 0.890, indicating substantial agreement (33), highlights the high level of consistency among human evaluators in assessing the quality and accuracy of AI-generated outputs. This robust inter-rater reliability suggests that evaluators share a common understanding of the evaluation criteria, reinforcing the reliability of the assessment process.

However, notable discrepancies in grading the “completeness” dimension reveal the influence of subjective biases inherent in guideline-based evaluations. These inconsistencies likely stem from differences in individual interpretation of guidelines, varying levels of domain expertise, or ambiguity in defining “completeness” within the context of AI outputs. Such subjectivity underscores the limitations of relying solely on human judgment for evaluating complex AI-generated content. To address these challenges, future studies could enhance the objectivity and reproducibility of evaluations by integrating automated adherence metrics. For instance, employing established semantic similarity indices, such as cosine similarity or BERT-based embeddings, to measure the alignment between AI outputs and guideline excerpts could provide a standardized, quantitative approach to assessing completeness (34, 35). These automated metrics would reduce reliance on subjective interpretation by offering a data-driven evaluation of how closely AI responses adhere to predefined guidelines. Additionally, incorporating natural language processing (NLP) techniques, such as topic modeling or keyword extraction, could further refine the evaluation process by identifying key thematic elements in both AI outputs and guidelines (36). Combining these automated tools with human oversight could create a hybrid evaluation framework that balances objectivity with the nuanced understanding that human evaluators bring.

This study conducted a comparative evaluation of text generated by four advanced large language models, ChatGPT-4o, DeepSeek-R1, Grok-3 and Claude-3.7, with a focus on their readability and applicability in healthcare communication. The assessment employed two well-established readability metrics: the FRE score, which measures text comprehensibility on a scale from 0 (extremely difficult) to 100 (very easy), and the FKGL, which estimates the U.S. school grade level required to understand the text (37, 38). The findings indicated that ChatGPT-4o-generated text was marginally more readable than DeepSeek-R1, based on both FRE scores and FKGL. Nonetheless, when evaluated against the standard FRE benchmark, all evaluated texts from the four models, received a classification of “extremely difficult”, defined by scores below 30. This suggests that, irrespective of the model used, the texts posed significant comprehension challenges for general readers, particularly patients or members of the public seeking accessible health information.

The FKGL results reinforced this conclusion, indicating that the texts required a reading proficiency equivalent to college-level education or higher, far exceeding the capabilities of typical lay audiences. The elevated reading difficulty was primarily attributed to the extensive incorporation of specialized medical terminology within the background prompts guiding the language models. These prompts were designed to ensure clinical accuracy and relevance, embedding domain-specific jargon which are standard in medical discourse but often unfamiliar to non-experts. While this technical language enhances the perceived precision and specificity of the content for clinical professionals such as physicians, nurses, or medical researchers, it creates a substantial cognitive barrier for lay readers. For example, patients or caregivers attempting to access health information may find such terminology confusing or intimidating, hindering their ability to make informed decisions about their care. In contrast, in clinical contexts where precision is critical, including the documentation of diagnostic rationales, treatment protocols, or research findings, the use of technical language is often essential to avoid ambiguity and ensure alignment with standardized medical guidelines.

This trade-off highlights a key tension: while technical rigor supports professional accuracy, it compromises accessibility for broader audiences, limiting the practical utility of AI-generated health content for non-specialist users. Based on this, a “clinical language dynamic adaptation module” can be developed, which can dynamically adjust the density of terms and the complexity of sentence structures by real-time identification of the user’s identity, such as patients, grassroots medical staff, or specialists, thereby achieving a balance between professional rigor and cognitive accessibility.

The study has several limitations. Firstly, the inherent stochasticity of generative AI outputs persists despite protocol-mandated dialogue resetting between queries. This variability stems from probabilistic decoding mechanisms inherent in transformer-based architectures and may affect response reproducibility across sessions.

Secondly, the exclusive use of English for prompts and data collection limits the generalizability of our results in multilingual settings and non-English-speaking populations. To address this constraint, subsequent research should adopt a tiered multilingual evaluation framework. We recommend beginning with languages prevalent among diabetic populations such as Spanish, Mandarin, and Arabic, with subsequent expansion to regional languages. This approach should include professional translation and back-translation of standardized diabetic foot infection scenarios, cultural adaptation of clinical contexts by native-speaking healthcare professionals, and comprehensive assessment of both linguistic accuracy and cultural appropriateness in model responses. Such a framework would incorporate metrics for medical terminology consistency, conceptual equivalence, and practical applicability across diverse healthcare environments.

Furthermore, the study does not evaluate the impact of model fine-tuning or domain-specific adaptation on performance. General-purpose LLMs may struggle with specialized medical terminology or nuanced patient-provider interactions without targeted optimization. The absence of such adaptations could potentially skew readability metrics including FRE score and FKGL, leading to inaccurate estimates of text comprehensibility in real-world clinical scenarios.

Finally, the dynamic nature of AI models necessitates systematic long-term performance tracking. We propose a structured framework involving quarterly assessments across three key dimensions: guideline adherence using standardized clinical vignettes, temporal consistency in response quality through longitudinal analysis of identical queries, and adaptation to evolving medical evidence via dynamic benchmarking against updated IWGDF/IDSA guidelines.

Beyond such technical assessments, clinical validation represents an essential next step. Randomized controlled trials comparing LLM-assisted decision-making with standard care should examine patient-centered outcomes including diagnostic accuracy, antibiotic selection appropriateness, time to infection resolution, and patient satisfaction metrics. Concurrent evaluation of implementation challenges, particularly workflow integration and clinician acceptance, will be crucial for establishing the practical utility of AI-assisted approaches in diabetic foot infection management.

## Conclusion

This study has confirmed the potential of tools such as ChatGPT-4o, DeepSeek-R1, Grok-3, and Claude-3.7 as auxiliary tools for the management of diabetic foot lesions. All models demonstrated high accuracy in meeting the standards of the International Working Group on Diabetic Foot/Guidelines of the American Diabetes Association. However, Grok-3 provided more contextual information and richer content in its responses. The text readability of all four models was very challenging, especially DeepSeek-R1, which might be more suitable for professional users who require detailed and domain-specific content. With the development of LLMs, they show potential as clinical auxiliary tools in the field of DFI management, but careful prompt design and domain validation remain crucial.

Furthermore, we propose the directions for future research. These include long-term performance tracking, multilingual evaluations, and model adaptations for healthcare contexts. In conclusion, while AI models show promise in supporting DFI management, their integration into clinical practice requires careful validation, human oversight, and strategies to improve accessibility for diverse audiences to ensure effective and equitable healthcare communication.

## Data Availability

The original contributions presented in the study are included in the article/**Supplementary Material**. Further inquiries can be directed to the corresponding authors.
