# Supplementary material for: Comparative assessment of large language models in diabetic foot infection management: alignment with IWGDF/IDSA guidelines
Source: Front Endocrinol (Lausanne). 2026 Feb 24;17:1667159. doi: 10.3389/fendo.2026.1667159 (PMC12971450; doi:10.3389/fendo.2026.1667159)
Supplement: Supplementary file 1 [file Table1.docx]

Supplementary Table 1 Summary of concordance between IWGDF/IDSA guidelines on the diagnosis and treatment of DFI and the responses from the AI models

| No. | Question and responses |
| --- | --- |
| 1 | Can the International Working Group of the Diabetic Foot/IDSA(IWGDF/IDSA) classification system for foot infections in persons with diabetes predict the outcome of such an infection？  **ChatGPT-4o response:**  Yes, the International Working Group on the Diabetic Foot (IWGDF) and the Infectious Diseases Society of America (IDSA) classification system for diabetic foot infections can help predict the outcome of these infections. The classification system is designed to assess the severity of foot infections in diabetic patients, primarily focusing on the depth and extent of the infection.  This classification system evaluates factors like:  1.Infection depth: Whether the infection is superficial, deep, or involving bone.  2.Extent of infection: Whether the infection is localized or spreading.  3.Clinical signs: Severity of symptoms, such as fever, erythema, or drainage.  4.Risk of complications: The likelihood of osteomyelitis or sepsis.  By classifying the infection, healthcare providers can determine the appropriate treatment plan (e.g., antibiotics, surgical intervention) and monitor the risk of complications. The severity of the infection as determined by the classification system is correlated with the likelihood of successful outcomes, including wound healing and the risk of amputation.  In summary, the IWGDF/IDSA classification system plays a crucial role in guiding treatment decisions and predicting clinical outcomes in diabetic foot infections. However, clinical outcomes also depend on other factors, including the patient's overall health, comorbid conditions, and the timeliness of treatment.  **DeepSeek-R1 response:**  The IWGDF/IDSA classification system for diabetic foot infections (DFIs) is primarily designed to stratify infection severity (mild, moderate, severe) and guide treatment decisions. While it is not explicitly validated as a standalone predictor of clinical outcomes (e.g., healing time, amputation risk, or mortality), its structured approach to assessing infection severity indirectly correlates with prognosis. Here’s a synthesis of insights from the search results:  Severity-Based Stratification and Clinical Guidance The system categorizes infections based on clinical signs (e.g., erythema, purulence, systemic inflammation) and anatomical involvement. For example:  Mild infections (localized) are managed with oral antibiotics and outpatient care, often leading to better outcomes with timely intervention.  Severe infections (systemic toxicity or limb-threatening features) require hospitalization, aggressive debridement, and intravenous antibiotics, which are linked to higher risks of amputation or mortality. This stratification aligns with the 2019 IWGDF guidelines, which emphasize tailored management based on infection severity.  Practical Use During COVID-19 During the pandemic, the IWGDF/IDSA system was adapted for remote triage. Patients with mild infections were managed via telemedicine, while severe cases were prioritized for urgent care. Outcomes depended on adherence to this risk stratification, suggesting the system’s utility in predicting resource allocation needs and potential complications.  Limitations in Predictive Accuracy The system focuses on clinical presentation but does not fully account for comorbidities (e.g., peripheral artery disease) or social determinants (e.g., access to care), which significantly influence outcomes. For instance, a patient with a "moderate" infection but untreated ischemia may still face high amputation risk despite classification.  Integration with Multidisciplinary Care The IWGDF stresses multidisciplinary teams (MDTs) for optimal outcomes. The classification system serves as a framework for MDTs to combine infection severity with vascular, neurological, and microbiological data, enhancing prognostic accuracy.  Evidence from Guidelines The 2019 IWGDF guidelines highlight that infection severity, as classified by IWGDF/IDSA, correlates with healing rates and complications. However, they caution that additional factors (e.g., glycemic control, wound offloading) must be addressed to improve predictive models.  Conclusion  The IWGDF/IDSA classification system is a valuable tool for risk stratification and guiding treatment, which indirectly informs prognosis. While it does not directly predict outcomes like a scoring system, its integration into comprehensive care pathways improves clinical decision-making and resource allocation. Further research is needed to validate its predictive power in diverse populations.  **Grok-3 response:**  The International Working Group on the Diabetic Foot (IWGDF) and Infectious Diseases Society of America (IDSA) classification system for diabetic foot infections (DFIs) is a widely used framework to assess the severity of infections and guide treatment. It categorizes infections into mild, moderate, and severe based on clinical findings such as the presence of inflammation, wound depth, and systemic signs like fever or metabolic disturbances. The system also includes an "uninfected" category for wounds without signs of infection.  Predictive Value of the IWGDF/IDSA Classification System  The IWGDF/IDSA classification system has been validated as a tool to predict clinical outcomes of diabetic foot infections, particularly in terms of hospitalization, amputation, and other complications. Key evidence includes:  Validation Studies:  A longitudinal study of 1,666 persons with diabetes demonstrated that increasing infection severity, as classified by the IWGDF/IDSA system, was associated with a significantly higher risk of:  Amputation (χ² test for trend, 108.0; P < .001)  Higher-level amputation (χ² test for trend, 113.3; P < .001)  Lower extremity-related hospitalization (χ² test for trend, 118.6; P < .001)  In a 2-year cohort study, the system effectively predicted adverse outcomes, with severe infections linked to higher rates of hospitalization (89% for severe vs. 4% for mild) and amputation (70% for severe vs. 3% for mild).  Clinical Utility:  The classification system helps clinicians stratify patients based on infection severity, which informs decisions about hospitalization, antibiotic therapy, and surgical intervention. For example, mild infections are often managed outpatient with oral antibiotics, while moderate and severe infections typically require hospitalization and broader-spectrum antibiotics or surgery.  The system’s clear definitions and limited number of categories make it user-friendly for clinicians, enhancing its applicability in diverse healthcare settings.  Limitations and Proposed Refinements:  A 2020 study suggested that the IWGDF/IDSA system could better reflect outcomes if moderate and severe categories were merged and infections were stratified by soft tissue infection (STI) versus osteomyelitis. This study found that osteomyelitis patients had worse outcomes, including longer antibiotic duration (63.8 vs. 32.5 days), higher surgery frequency (99.4% vs. 55.5%), more amputations (83.4% vs. 26.3%), and longer hospital stays (22.6 vs. 14.5 days).  The 2023 IWGDF/IDSA guidelines reviewed whether the classification should be modified to combine moderate and severe categories or incorporate risk indicators like systemic inflammatory response syndrome (SIRS). However, due to insufficient high-quality evidence and moderate certainty attributed to study bias, no changes were made to the existing classification.  Current Recommendations:  The 2023 IWGDF/IDSA guidelines recommend using the classification scheme to assess DFI severity, emphasizing its role in clinical decision-making. However, they note that additional validation studies are needed to refine the system further, particularly for distinguishing outcomes in osteomyelitis cases or incorporating advanced diagnostic tools like molecular techniques.  Conclusion  The IWGDF/IDSA classification system is a validated and effective tool for predicting outcomes of diabetic foot infections, with strong evidence linking higher severity grades to increased risks of hospitalization, amputation, and other complications. While it is practical and widely applicable, ongoing research suggests potential refinements, such as stratifying by osteomyelitis, to improve its predictive accuracy. Clinicians should continue to use this system as part of a multidisciplinary approach to optimize patient outcomes.  For further details, refer to the 2023 IWGDF/IDSA guidelines at [https://www.idsociety.org](https://www.idsociety.org/) or [https://iwgdfguidelines.org](https://iwgdfguidelines.org/).  **Claude-3.7 response:**  The IWGDF/IDSA classification system for diabetic foot infections does have some predictive value for outcomes, but with important limitations.  The classification system categorizes infections into:  Mild infections (superficial, limited signs of inflammation)  Moderate infections (deeper or more extensive)  Severe infections (systemic signs or metabolic instability)  Research has shown that this classification correlates with certain outcomes:  Predictive strengths:  Higher severity grades generally associate with longer treatment duration, increased hospitalization rates, and greater likelihood of requiring surgical intervention.  Severe infections have significantly higher rates of amputation and mortality compared to mild infections.  The system helps guide initial antibiotic selection and treatment intensity.  Limitations in outcome prediction:  The classification doesn't account for important prognostic factors like vascular status, extent of tissue necrosis, bone involvement, or patient comorbidities.  Individual patient outcomes can vary significantly within the same classification category.  It's primarily designed for initial assessment and treatment guidance rather than precise outcome prediction.  Clinical utility: The system works best as a starting point for risk stratification and treatment planning, but clinicians typically need to consider additional factors like:  Perfusion status  Presence of osteomyelitis  Patient's overall health status  Social factors affecting adherence  While the IWGDF/IDSA classification provides valuable prognostic information and helps standardize care, it should be used alongside comprehensive clinical assessment rather than as a standalone outcome predictor. |
| 2 | In a person with diabetes and infection of the foot, which test(s) can best identify the causative pathogen(s), and result in tailored use of antibiotics？  **ChatGPT-4o response:**  In a person with diabetes and a foot infection, the following tests can help identify the causative pathogen(s) and guide tailored antibiotic therapy:  1.Wound Culture: A sample is taken from the infected area and cultured to identify the specific bacteria or fungi causing the infection. This is the most direct way to determine the pathogen and its antibiotic sensitivities.  2.Gram Staining: This can be done on wound exudate to quickly identify whether the infection is caused by Gram-positive, Gram-negative bacteria, or fungi, aiding in initial treatment decisions.  3.Blood Cultures: If there are signs of systemic infection, blood cultures can help detect the presence of bacteria in the bloodstream (bacteremia), which is critical for severe infections like sepsis.  4.X-ray or MRI: These imaging tests can identify deeper infections or bone involvement (osteomyelitis), which may require specific management and antibiotic choices.  5.Polymerase Chain Reaction (PCR): For identifying specific pathogens, including those that are difficult to culture (e.g., certain bacteria or fungi), PCR can be very useful.  By using these tests, healthcare providers can determine the specific pathogen and its antibiotic sensitivity, allowing for more precise and effective treatment.  **DeepSeek-R1 response:**  In a diabetic foot infection, identifying the causative pathogen(s) requires a combination of appropriate sampling and diagnostic methods to ensure targeted antibiotic therapy. Here’s a structured approach:  1. Specimen Collection  Deep tissue biopsy or curettage (after debridement of necrotic tissue) is gold standard. Avoid superficial swabs, which may capture colonizers or contaminants.  Bone biopsy if osteomyelitis is suspected (imaging-guided if needed).  2. Microbiological Tests  Aerobic and anaerobic cultures:  Identify common pathogens (e.g., Staphylococcus aureus, Streptococcus spp., Enterobacteriaceae, Pseudomonas aeruginosa, anaerobes).  Detect polymicrobial infections (common in diabetic ulcers).  Provide antibiotic susceptibility testing (AST) to guide therapy.  Gram stain: Rapid preliminary results to guide empiric therapy.  3. Molecular Techniques (if available, for complex cases):  PCR/multiplex PCR panels:  Rapid detection of pathogens (e.g., MRSA via mecA gene) or resistance genes (e.g., ESBL, carbapenemases).  Useful if prior antibiotics may suppress culture growth.  16S rRNA sequencing: For culture-negative cases or atypical infections.  4. Adjunctive Tests  Blood cultures: If systemic signs of infection (e.g., fever, sepsis) are present.  MALDI-TOF mass spectrometry: Accelerates pathogen identification from positive cultures.  5. Key Considerations  Avoid empiric broad-spectrum antibiotics without cultures to prevent resistance.  Local antibiograms should guide empiric therapy while awaiting results.  Repeat cultures if clinical failure occurs.  Conclusion  Deep tissue/bone cultures with AST remain the cornerstone for tailored antibiotic use. Molecular methods (PCR, sequencing) supplement complex cases. Proper sampling technique is critical to avoid misleading results.  **Grok-3 response:**  To identify the causative pathogen(s) in a diabetic foot infection and guide tailored antibiotic therapy, the following tests are most effective:  Deep Tissue Culture:  Description: Obtaining a sample from deep tissue or bone (via biopsy or debridement) is the gold standard for identifying pathogens. Swabs from superficial wounds are less reliable due to contamination with skin flora.  Why it’s effective: Cultures from deep tissue or bone accurately identify the causative bacteria, including anaerobes or polymicrobial infections common in diabetic foot infections. This helps select targeted antibiotics.  Process: After debridement, a sample is sent for aerobic and anaerobic bacterial cultures, and sometimes fungal or mycobacterial cultures if clinically indicated.  Wound Swab Culture (if deep tissue not feasible):  Description: A swab from the wound base after cleaning and debridement can be used, though it’s less reliable than tissue culture.  Why it’s effective: It can still identify dominant pathogens, but results must be interpreted cautiously due to potential contamination.  Blood Cultures:  Description: Indicated in cases of systemic infection (e.g., sepsis or fever).  Why it’s effective: Identifies bloodstream pathogens, which may correlate with the foot infection, aiding in antibiotic selection.  Molecular Testing (PCR or Next-Generation Sequencing):  Description: Polymerase chain reaction (PCR) or next-generation sequencing (NGS) can detect bacterial DNA, including resistant strains.  Why it’s effective: These tests are highly sensitive and can identify pathogens in culture-negative cases or detect resistance genes (e.g., MRSA), guiding precise antibiotic therapy.  Imaging-Guided Biopsy (if osteomyelitis is suspected):  Description: If imaging (e.g., MRI or X-ray) suggests bone involvement, a bone biopsy for culture is critical.  Why it’s effective: Osteomyelitis in diabetic foot infections often requires prolonged, targeted antibiotic therapy based on bone culture results.  Additional Considerations:  Antibiotic Sensitivity Testing: Once pathogens are identified, antibiotic susceptibility testing (e.g., via Kirby-Bauer disk diffusion or MIC testing) is essential to tailor therapy, especially given the high risk of multidrug-resistant organisms (e.g., MRSA, Pseudomonas) in diabetic foot infections.  Common Pathogens: Expect polymicrobial infections, including Staphylococcus aureus (including MRSA), Streptococcus spp., Enterococcus, Pseudomonas aeruginosa, and anaerobes like Bacteroides.  Empiric Therapy: While awaiting culture results, empiric antibiotics (e.g., vancomycin, piperacillin-tazobactam, or carbapenems) may be started based on local resistance patterns and infection severity, later adjusted based on culture results.  Recommendation: Deep tissue or bone biopsy cultures, combined with antibiotic sensitivity testing, are the most reliable for identifying pathogens and guiding tailored antibiotic therapy in diabetic foot infections. Molecular testing can complement these when available, especially for complex or resistant cases. Always correlate results with clinical findings and consult infectious disease specialists for severe infections.  **Claude-3.7 response:**  For diabetic foot infections, the most effective approach to identify causative pathogens and guide antibiotic therapy involves:  Primary diagnostic tests:  Deep tissue culture is the gold standard. This involves obtaining tissue samples from the base of a debrided ulcer or through needle aspiration of purulent material. Deep tissue cultures are superior to superficial swab cultures because they better represent the actual infecting organisms rather than surface colonizers.  Bone biopsy with culture should be performed when osteomyelitis is suspected, as this provides the most accurate identification of pathogens causing bone infection.  Supporting tests:  Blood cultures are important when systemic infection is suspected, though they're often negative in localized diabetic foot infections.  Molecular diagnostics like PCR-based assays can be valuable adjuncts, especially for detecting fastidious organisms or when prior antibiotic use may affect culture results.  Key considerations for optimal results:  Samples should be obtained before starting antibiotics when possible  Proper debridement of necrotic tissue before sampling improves accuracy  Both aerobic and anaerobic cultures should be requested  Gram staining provides rapid preliminary information  Common pathogens in diabetic foot infections:  Mild infections: Usually Staphylococcus aureus and Streptococcus species  Moderate to severe infections: Often polymicrobial, including gram-positive cocci, gram-negative bacilli, and anaerobes.  The combination of deep tissue culture with appropriate clinical assessment allows for targeted antibiotic therapy rather than broad-spectrum empirical treatment, improving outcomes while reducing antibiotic resistance risks. |
| 3 | In a person with diabetes and suspected bone or joint infection of the foot, which tests have the best correlation with BonE BiOPsy(BeBoP) results for diagnosing diabetes‐related osteomyelitis, including residual/postoperative osteomyelitis)？  **ChatGPT-4o response:**  In diagnosing diabetes-related osteomyelitis, particularly in patients with suspected bone or joint infection of the foot, the following tests have been shown to have the best correlation with bone biopsy (BeBoP) results:  1.Magnetic Resonance Imaging (MRI):  MRI is considered one of the most accurate non-invasive imaging methods for diagnosing osteomyelitis. It is highly sensitive in detecting bone marrow changes and soft tissue involvement, which are characteristic of osteomyelitis.  MRI findings such as increased signal intensity on T2-weighted images and bone marrow edema suggest infection and have a high correlation with biopsy results.  2.Bone Scintigraphy (Bone Scan):  Bone scans using technetium-99m-labeled radiopharmaceuticals are sensitive for detecting osteomyelitis but are less specific. A positive result in the context of diabetic foot infection can indicate the presence of osteomyelitis, but further confirmatory tests, such as bone biopsy, are often needed.  3.Computed Tomography (CT) Scan:  CT scans can provide detailed images of bone structures and are useful in detecting chronic osteomyelitis with sequestra or bone destruction. However, CT is not as sensitive as MRI in detecting early infection.  4.Procalcitonin (PCT) and C-Reactive Protein (CRP):  While these biomarkers are not definitive for osteomyelitis, they are useful in the assessment of infection. Elevated levels of procalcitonin and CRP can indicate the presence of infection, but these markers alone are not specific to osteomyelitis.  5.Blood Cultures:  Blood cultures can be useful if there is a suspicion of systemic infection, but they may not always detect bone infection, especially in cases of localized diabetic foot osteomyelitis.  6.Bone Biopsy (BeBoP):  While bone biopsy remains the gold standard for diagnosing osteomyelitis, it is invasive and carries risks. It is often performed when non-invasive tests like MRI or bone scans strongly suggest osteomyelitis, but the diagnosis remains uncertain.  In summary, MRI is the best non-invasive test that correlates strongly with bone biopsy results for diagnosing osteomyelitis in diabetic foot infections, especially in the case of postoperative or residual osteomyelitis. Bone scintigraphy and CT scans can also be helpful, while biomarkers like CRP and procalcitonin support the clinical picture but are not definitive for osteomyelitis.  **DeepSeek-R1 response:**  In diagnosing diabetes-related osteomyelitis (including residual or postoperative cases) in the foot, several tests correlate well with bone biopsy (BeBoP) results. Here's a synthesis of the evidence-based recommendations from the provided search results:  1. 18-FDG-PET/CT (Fluorodeoxyglucose Positron Emission Tomography/Computed Tomography)  High Diagnostic Accuracy: 18-FDG-PET/CT demonstrates excellent sensitivity (90–94%) and specificity (87–93%) for detecting osteomyelitis, closely aligning with bone biopsy results.  Advantages in Complex Cases: It is particularly effective in differentiating osteomyelitis from Charcot neuro-osteoarthropathy and assessing residual/postoperative infections due to its ability to detect metabolic activity in infected bone.  Guideline Support: Recommended by the European Association of Nuclear Medicine (EANM) for evaluating diabetic foot infections, especially when conventional imaging is inconclusive.  2. MRI (Magnetic Resonance Imaging)  High Sensitivity and Specificity: MRI is highly sensitive (90–100%) and specific (75–85%) for detecting osteomyelitis. It provides detailed anatomical visualization of bone marrow edema, abscesses, and sinus tracts.  Limitations in Postoperative Settings: While effective, MRI may be less reliable post-surgery due to artifacts from metal implants or residual inflammation, reducing its correlation with biopsy results in these cases.  3. Leukocyte Scintigraphy (White Blood Cell Scanning)  Targeted Imaging: Radionuclide scans using labeled leukocytes (e.g., technetium-99m) show moderate accuracy (sensitivity 80–85%, specificity 70–75%) for osteomyelitis. They are useful for distinguishing soft tissue infection from bone involvement.  Combined Protocols: Combining leukocyte scans with bone marrow imaging improves specificity by reducing false positives from non-infectious inflammation.  4. Probe-to-Bone Test and Serum Biomarkers  Clinical Utility: The probe-to-bone test (positive predictive value ~85%) and elevated inflammatory markers (e.g., erythrocyte sedimentation rate [ESR] >70 mm/h) are cost-effective initial screens. When combined, their diagnostic accuracy improves.  Limitations: These tests lack the specificity of imaging or biopsy and are more useful for triaging patients to advanced diagnostics.  5. Microbiological Culture and Histopathology  Gold Standard Correlation: While bone biopsy remains definitive, deep tissue cultures and histopathology of surgical specimens (e.g., debrided bone) align closely with biopsy results. However, contamination risks and prior antibiotic use may reduce accuracy.  Key Recommendations  First-Line Imaging: Use 18-FDG-PET/CT or MRI for high accuracy, especially in complex or postoperative cases.  Adjunctive Tests: Combine imaging with serum biomarkers (ESR, CRP) and clinical evaluation (probe-to-bone) to enhance diagnostic confidence.  Biopsy When Uncertain: Proceed to bone biopsy if imaging and clinical findings are discordant or inconclusive.  For residual/postoperative osteomyelitis, 18-FDG-PET/CT is particularly advantageous due to its ability to bypass surgical artifacts and distinguish active infection from healing tissue. Always integrate results with multidisciplinary clinical judgment for optimal outcomes.  **Grok-3 response:**  Diagnosing diabetes-related osteomyelitis, including residual or postoperative cases, relies on tests that correlate well with bone biopsy (BeBoP), the gold standard. Based on available evidence, the following tests show the strongest correlation with BeBoP results:  Magnetic Resonance Imaging (MRI):  Correlation with BeBoP: MRI has the highest diagnostic accuracy among imaging modalities, with sensitivity of 90–100% and specificity of 80–90% for detecting osteomyelitis. It excels in identifying bone marrow edema, cortical destruction, and soft tissue involvement, which align closely with biopsy-confirmed infection.  Strengths: MRI is particularly effective for detecting early and residual osteomyelitis, as it visualizes bone and soft tissue changes not seen on other imaging. T1-weighted images showing low signal intensity in bone marrow and T2-weighted images with high signal intensity are highly suggestive of osteomyelitis.  Limitations: May be less specific in postoperative cases due to surgical changes mimicking infection. Metallic implants can cause artifacts.  Probe-to-Bone (PTB) Test:  Correlation with BeBoP: The PTB test has a positive predictive value (PPV) of ~85–95% in high-prevalence settings (e.g., diabetic foot ulcers with suspected infection). A positive PTB (palpating bone with a sterile probe through an ulcer) strongly correlates with biopsy-confirmed osteomyelitis.  Strengths: Simple, bedside test with high specificity in chronic wounds. It’s particularly useful in resource-limited settings.  Limitations: Lower sensitivity (~60–70%), so a negative test doesn’t rule out osteomyelitis. Less reliable in postoperative settings due to altered anatomy.  Bone Scan with Labeled White Blood Cell (WBC) Imaging:  Correlation with BeBoP: Labeled WBC scans (e.g., indium-111 or technetium-99m) have a sensitivity of 80–90% and specificity of 70–80% for osteomyelitis. They correlate well with biopsy by detecting active infection through leukocyte accumulation.  Strengths: Useful for confirming infection in complex cases, including residual or postoperative osteomyelitis, where MRI is inconclusive.  Limitations: Time-consuming, expensive, and less specific in the presence of inflammation (e.g., Charcot foot or recent surgery).  Erythrocyte Sedimentation Rate (ESR) and C-Reactive Protein (CRP):  Correlation with BeBoP: Elevated ESR (>70 mm/h) and CRP (>10 mg/L) have moderate correlation with biopsy-proven osteomyelitis, with sensitivity of 80–90% but lower specificity (~50–60%). Persistent elevation post-surgery may indicate residual infection.  Strengths: Non-invasive, widely available, and useful for monitoring treatment response.  Limitations: Non-specific, as levels can be elevated in soft tissue infection or other inflammatory conditions.  X-ray:  Correlation with BeBoP: X-rays have lower sensitivity (50–60%) and specificity (60–70%) compared to MRI but can show chronic osteomyelitis features (e.g., periosteal reaction, cortical erosion) that align with biopsy findings in advanced cases.  Strengths: Inexpensive and widely available. Useful for detecting postoperative changes like hardware loosening.  Limitations: Early or residual osteomyelitis may be missed, as changes take weeks to appear.  Summary:  Best Overall: MRI is the most reliable non-invasive test due to its high sensitivity and specificity, closely correlating with BeBoP for both initial and residual osteomyelitis.  Practical Bedside Option: PTB test is highly specific in chronic wounds and correlates well with biopsy in high-risk cases.  Adjunctive Tests: WBC scans and inflammatory markers (ESR/CRP) provide supportive evidence, especially in complex or postoperative cases, but are less definitive alone.  Caveat: No test matches BeBoP’s accuracy, as imaging and biomarkers can be confounded by non-infectious conditions (e.g., Charcot foot, postsurgical changes). Combining clinical assessment, MRI, and PTB offers the best non-invasive diagnostic approach.  If you need a deeper dive into specific study data or test protocols, let me know!  **Claude-3.7 response:**  For diagnosing diabetes-related osteomyelitis in the foot, several tests show varying degrees of correlation with bone biopsy results. Here's what the evidence shows:  Imaging Studies  MRI demonstrates the strongest correlation with bone biopsy results. It has high sensitivity (85-95%) and good specificity (70-85%) for detecting osteomyelitis. MRI can distinguish between soft tissue infection and bone involvement, and can identify bone marrow edema, cortical destruction, and surrounding soft tissue changes that correlate well with histopathologic findings.  Plain radiographs have limited correlation with early osteomyelitis, as they require 30-50% bone destruction before changes become visible. However, when positive, they show good correlation with advanced disease found on biopsy.  Nuclear medicine studies (bone scintigraphy, labeled white blood cell scans) have moderate correlation but can be challenging to interpret in diabetic patients due to concurrent soft tissue infection and neuropathic changes.  Laboratory Markers  ESR and CRP show poor to moderate correlation with bone biopsy results. While elevated inflammatory markers support the diagnosis, they can be elevated due to soft tissue infection alone and may be normal in chronic osteomyelitis.  Procalcitonin has shown promise in some studies but evidence is still limited for routine use in diabetic foot osteomyelitis.  Clinical Assessment  The probe-to-bone test (ability to probe to bone through a foot ulcer) shows good correlation with osteomyelitis on biopsy, particularly when the ulcer is >2 cm² or when bone is easily palpable.  Key Considerations  Tissue culture from bone biopsy remains the gold standard for identifying causative organisms  Histopathologic examination of bone samples provides definitive diagnosis.  Combined clinical, laboratory, and imaging approaches typically provide better correlation than any single test.  MRI combined with clinical assessment (including probe-to-bone testing when applicable) generally provides the best non-invasive correlation with bone biopsy results for diagnosing diabetes-related foot osteomyelitis. |
| 4 | In a person with diabetes and a soft‐tissue infection of the foot, which specific antibiotic regimen (specific agent[s], route of administration, duration of therapy) should be chosen when taking into account the resolution and recurrence of infection, and the acquisition of antimicrobial resistance？  **ChatGPT-4o response:**  In a person with diabetes and a soft-tissue infection of the foot, the choice of antibiotic regimen depends on the severity of the infection, local resistance patterns, and the need to address common pathogens associated with diabetic foot infections (DFI), such as Staphylococcus aureus (including methicillin-resistant S. aureus [MRSA]), Streptococcus species, and Gram-negative bacteria.  Here is a general approach for antibiotic selection, tailored to minimize resistance and ensure effective resolution:  1. Mild to Moderate Infection (Uncomplicated)  Common Pathogens: Streptococcus spp., S. aureus (including MRSA), Enterobacteriaceae (E. coli).  Initial Empiric Therapy (Oral agents):  Cephalexin (500 mg every 6 hours) or Amoxicillin-Clavulanate (875 mg/125 mg every 12 hours) — effective for Gram-positive cocci and some Gram-negative organisms.  Clindamycin (300-450 mg every 6-8 hours) — a good option if MRSA is suspected, though less effective against Gram-negative organisms.  Doxycycline (100 mg every 12 hours) or Trimethoprim-Sulfamethoxazole (1-2 double-strength tablets every 12 hours) — alternatives if MRSA is a concern.  Duration: 5-14 days, depending on clinical improvement.  2. Severe Infection (Complicated or Systemic Symptoms)  Common Pathogens: More extensive involvement of Gram-negative bacteria (including Pseudomonas spp.), MRSA, and anaerobes.  Initial Empiric Therapy (Intravenous therapy, if systemic infection or severe soft tissue infection):  Vancomycin (15 mg/kg every 12 hours) — for MRSA coverage.  Piperacillin-Tazobactam (3.375 g every 6 hours) — broad-spectrum coverage, including Gram-positive, Gram-negative, and anaerobes.  Alternatively, Ceftriaxone (2 g every 24 hours) with Metronidazole (500 mg every 8 hours) — if anaerobic infection is a concern.  For pseudomonal coverage: Cefepime (2 g every 8 hours) or Meropenem (1 g every 8 hours) may be required.  Duration: 7-14 days based on clinical response and severity of the infection.  3. Monitoring and Adjusting Therapy  Clinical Response: After 48-72 hours, assess the patient’s clinical improvement. If there is improvement, consider switching from IV to oral therapy.  Culture and Sensitivity: If possible, obtain a culture of the wound to tailor antibiotic therapy based on specific bacterial pathogens and resistance profiles.  4. Prevention of Recurrence and Resistance  Infection Control: Proper management of underlying diabetes, including strict blood sugar control, is crucial in preventing infection recurrence.  Adherence to Therapy: Educate patients on completing the full course of antibiotics to prevent resistance.  Prevention: Consider prophylactic antibiotics only in high-risk patients (those with prior infections, poor circulation, or immunosuppressed conditions).  Conclusion:  Mild to Moderate: Oral agents such as cephalexin or amoxicillin-clavulanate.  Severe or Systemic Infections: Intravenous vancomycin plus piperacillin-tazobactam or cefepime with metronidazole.  Duration: Typically 5-14 days for uncomplicated cases, and 7-14 days for complicated cases.  It’s important to individualize therapy based on the patient’s specific circumstances, including the severity of infection, comorbidities, and the results of microbiological cultures.  **DeepSeek-R1 response:**  Antibiotic Regimen for Diabetic Soft-Tissue Foot Infection Key Considerations: Severity of infection, local resistance patterns, MRSA risk, and culture-guided de-escalation to minimize resistance and recurrence.  1. Classification and Initial Empiric Therapy  Mild Infection (superficial, no systemic symptoms, low MRSA risk):  Oral Regimen: Amoxicillin/clavulanate (875/125 mg every 12 hours) for 1–2 weeks.  MRSA Coverage (if risk factors): Add TMP-SMX (1–2 DS tablets every 12 hours) or doxycycline (100 mg every 12 hours).  Moderate to Severe Infection (deep/extensive, systemic signs, or high MRSA risk):  IV Regimen:  Broad Gram-negative + Anaerobe Coverage: Piperacillin/tazobactam (3.375 g every 6 hours) or ceftriaxone (2 g daily) + metronidazole (500 mg every 8 hours).  MRSA Coverage: Add vancomycin (15–20 mg/kg every 8–12 hours, adjusted for renal function) or linezolid (600 mg every 12 hours).  2. Culture-Guided Adjustment  De-escalate based on culture results (e.g., narrow spectrum for susceptible organisms).  MRSA-Positive: Transition to oral TMP-SMX or doxycycline if improving.  No MRSA: Stop anti-MRSA agents to reduce resistance risk.  3. Route Transition  IV to Oral Switch: After 3–5 days of clinical improvement (e.g., reduced inflammation, afebrile).  Options: Amoxicillin/clavulanate (if susceptible) or clindamycin (if Gram-positive coverage needed) + TMP-SMX (for Gram-negative/MRSA).  4. Duration  Mild: 1–2 weeks (until clinical resolution).  Moderate/Severe: 2–4 weeks (extend if slow response; avoid overlong courses to limit resistance).  5. Adjunctive Measures  Surgical Intervention: Debridement for abscess/necrosis.  Wound Care: Offloading, glycemic control, and vascular assessment.  6. Resistance/Recurrence Prevention  Avoid Broad-Spectrum Overuse: De-escalate promptly.  Adherence: Ensure completion of prescribed regimen.  Follow-Up: Monitor for recurrence and adjust therapy if cultures reveal resistance.  Guideline Alignment: Aligns with IDSA recommendations, emphasizing severity stratification, MRSA risk assessment, and targeted therapy to balance efficacy with antimicrobial stewardship.  **Grok-3 response:**  Choosing an antibiotic regimen for a person with diabetes and a soft-tissue infection of the foot requires careful consideration of factors such as the severity of the infection, likely pathogens, local antimicrobial resistance patterns, patient-specific factors (e.g., renal function, allergies), and the goals of resolving the infection, preventing recurrence, and minimizing antimicrobial resistance. Below is a general approach based on current clinical guidelines and evidence, while acknowledging that specific recommendations should be tailored by a healthcare provider based on culture results, local resistance patterns, and patient characteristics.  Key Considerations  Pathogens: Diabetic foot infections (DFIs) are often polymicrobial. Common pathogens include:  Gram-positive cocci: Staphylococcus aureus (including methicillin-resistant S. aureus [MRSA]), Streptococcus species.  Gram-negative bacilli: Pseudomonas aeruginosa, Escherichia coli, Klebsiella spp., especially in chronic or severe infections.  Anaerobes: In deep or necrotic wounds (e.g., Bacteroides spp., Peptostreptococcus).  Severity: DFIs are classified as mild, moderate, or severe (e.g., per the Infectious Diseases Society of America [IDSA] guidelines). This impacts the choice of antibiotics, route, and duration.  Antimicrobial Resistance: Overuse of broad-spectrum antibiotics can drive resistance. Targeted therapy based on culture and susceptibility testing is preferred to minimize resistance.  Recurrence: Recurrence is common in DFIs due to underlying factors like neuropathy, poor wound healing, and vascular insufficiency. Optimal antibiotic choice, combined with wound care and offloading, reduces recurrence risk.  General Antibiotic Regimens by Severity  The following are general recommendations based on IDSA 2012 guidelines (still widely referenced) and more recent literature up to 2025. Always obtain cultures (preferably deep tissue or bone if osteomyelitis is suspected) to guide therapy.  1. Mild Infections  Characteristics: Superficial, limited cellulitis (<2 cm), no systemic symptoms, no deep tissue involvement.  Likely Pathogens: Primarily S. aureus and Streptococcus spp.  Antibiotic Regimen:  Agent(s):  Oral options:  Dicloxacillin 500 mg q6h or cephalexin 500 mg q6h (covers MSSA and Streptococcus).  If MRSA is suspected (e.g., prior history, local prevalence): Clindamycin 300–450 mg q6–8h or doxycycline 100 mg q12h.  Alternative for MRSA: Trimethoprim-sulfamethoxazole (TMP-SMX) 1–2 DS tablets q12h.  Route: Oral.  Duration: 7–14 days, until clinical resolution. Extend if slow to resolve but avoid unnecessarily prolonged courses to reduce resistance risk.  Rationale: Narrow-spectrum agents targeting gram-positive organisms are sufficient for mild infections. MRSA coverage depends on risk factors (e.g., prior MRSA, non-healing wounds).  2. Moderate Infections  Characteristics: Deeper infection, cellulitis >2 cm, lymphangitis, or abscess, but no systemic inflammatory response syndrome (SIRS).  Likely Pathogens: Polymicrobial, including gram-positive cocci, gram-negative bacilli, and possibly anaerobes.  Antibiotic Regimen:  Agent(s):  Oral options (if stable, outpatient management feasible):  Amoxicillin-clavulanate 875/125 mg q12h or levofloxacin 750 mg q24h + clindamycin 300–450 mg q6–8h (for anaerobe coverage).  If MRSA suspected: TMP-SMX 1–2 DS tablets q12h or doxycycline 100 mg q12h + amoxicillin-clavulanate.  Intravenous (IV) options (if hospitalization or poor oral absorption):  Ceftriaxone 1–2 g q24h + metronidazole 500 mg q8h (for anaerobes).  If MRSA suspected: Vancomycin 15–20 mg/kg q12h (target trough 15–20 mg/L) or linezolid 600 mg q12h (IV or oral).  Route: Oral for stable outpatients; IV for hospitalized patients or those with poor oral absorption.  Duration: 1–2 weeks, depending on clinical response. Switch to oral therapy once improving to complete the course.  Rationale: Broader-spectrum agents are needed due to polymicrobial risk. Culture-guided therapy helps narrow the regimen, reducing resistance pressure.  3. Severe Infections  Characteristics: Systemic symptoms (fever, sepsis), deep tissue involvement, or osteomyelitis.  Likely Pathogens: Polymicrobial, including MRSA, gram-negative bacilli (e.g., Pseudomonas), and anaerobes.  Antibiotic Regimen:  Agent(s):  IV options:  Vancomycin 15–20 mg/kg q12h + piperacillin-tazobactam 4.5 g q6–8h (broad-spectrum, including Pseudomonas and anaerobes).  Alternative: Vancomycin + meropenem 1 g q8h (for multidrug-resistant gram-negatives).  If MRSA confirmed: Consider linezolid 600 mg q12h (IV/oral) or daptomycin 6 mg/kg q24h.  For suspected osteomyelitis: Add rifampin 300–600 mg q12h (if S. aureus) to enhance bone penetration, but only after initial control to avoid resistance.  Route: IV initially, with possible step-down to oral therapy (e.g., linezolid, fluoroquinolone + metronidazole) once stable.  Duration: 2–4 weeks for soft-tissue infection; 4–6 weeks or longer for osteomyelitis. Prolonged therapy increases resistance risk, so de-escalate based on cultures.  Rationale: Broad-spectrum IV therapy is critical initially due to life-threatening potential. De-escalation to targeted therapy based on cultures minimizes resistance.  Additional Considerations  Wound Care: Debridement, abscess drainage, and offloading are critical to reduce bacterial load and recurrence risk.  Antimicrobial Resistance: Obtain cultures before starting antibiotics to guide therapy. Avoid prolonged broad-spectrum antibiotics. Local antibiograms should inform empiric therapy.  Recurrence Prevention: Address underlying factors (e.g., glycemic control, vascular insufficiency). Recurrence is often due to poor wound healing rather than inadequate antibiotics.  Patient Factors:  Renal impairment: Adjust doses (e.g., vancomycin, levofloxacin).  Allergies: Avoid beta-lactams if allergic; use alternatives like clindamycin or doxycycline.  Monitoring: Regular follow-up to assess resolution, adjust therapy, and monitor for adverse effects (e.g., C. difficile with clindamycin).  Notes on Resistance and Recurrence  Resistance: Overuse of broad-spectrum agents (e.g., fluoroquinolones, carbapenems) increases resistance, particularly in gram-negative organisms and MRSA. Use culture-directed therapy and the narrowest spectrum possible.  Recurrence: Recurrence rates in DFIs are high (20–30% within 1–2 years). Antibiotics alone are insufficient; multidisciplinary care (podiatry, vascular surgery, endocrinology) is essential.  Final Notes  These recommendations are based on general principles from IDSA guidelines, recent literature, and clinical practice up to 2025. Specific regimens should be guided by:  Culture and sensitivity results.  Local resistance patterns (consult hospital or regional antibiograms).  Consultation with infectious disease specialists for complex cases.  If you need more specific guidance (e.g., for a particular patient profile or local resistance data), please provide additional details. For pricing or access to xAI products like Grok, visit [https://x.ai/grok](https://x.ai/grok).  **Claude-3.7 response:**  For diabetic foot soft-tissue infections, the antibiotic choice depends on infection severity and patient factors. Here's the evidence-based approach:  Mild to Moderate Infections (outpatient)  First-line oral options:  Clindamycin 300-450mg every 6-8 hours for 7-14 days  Trimethoprim-sulfamethoxazole 1-2 double-strength tablets twice daily for 7-14 days  Doxycycline 100mg twice daily for 7-14 days  These target common pathogens (Staphylococcus aureus, Streptococcus species) while minimizing resistance development.  Severe Infections (hospitalization required)  IV therapy initially, then oral step-down:  Vancomycin 15-20mg/kg every 8-12 hours (adjust for renal function) plus piperacillin-tazobactam 3.375g every 6 hours  Alternative: Linezolid 600mg every 12 hours (oral bioavailability allows earlier discharge)  Duration: 10-14 days total, with IV-to-oral transition once clinically stable (typically 3-5 days).  Key Considerations for Diabetic Patients  Antimicrobial resistance prevention:  Avoid fluoroquinolones as first-line due to rising resistance rates  Consider local antibiograms when available  Ensure adequate tissue penetration (clindamycin and linezolid have excellent soft-tissue penetration)  Diabetes-specific factors:  Monitor blood glucose closely (some antibiotics can affect glycemic control)  Assess renal function before dosing (common diabetic complication)  Consider wound culture to guide targeted therapy, especially for recurrent infections  Recurrence prevention:  Address underlying vascular compromise and glycemic control  Ensure complete treatment course compliance  Monitor for treatment failure requiring broader spectrum coverage  The goal is pathogen eradication while preserving future treatment options through judicious antibiotic selection and appropriate duration of therapy. |
| 5 | In a person with diabetes and a bone or joint infection of the foot, is any particular antibiotic regimen (specific agent[s], route of administration, total and parenteral duration) better than any other regarding the resolution and recurrence of infection？  **ChatGPT-4o response:**  In a person with diabetes and a bone or joint infection (osteomyelitis or septic arthritis) of the foot, antibiotic therapy must be chosen carefully to optimize resolution and prevent recurrence. The regimen often depends on the causative pathogens, the severity of the infection, and the patient's individual health status. Below is a general guideline, but treatment must always be tailored to the specific situation.  1. Causative Organisms  Common pathogens in diabetic foot infections include:  Gram-positive organisms: Staphylococcus aureus (including methicillin-resistant Staphylococcus aureus or MRSA), Streptococcus species.  Gram-negative organisms: Pseudomonas aeruginosa, Enterobacteriaceae.  Anaerobes: Bacteroides and Clostridium species, especially in polymicrobial infections.  2. Initial Empiric Antibiotic Therapy  Vancomycin or clindamycin (for MRSA coverage) combined with a beta-lactam (e.g., ceftriaxone or piperacillin-tazobactam) for broad coverage of gram-positive and gram-negative organisms.  Cefepime or meropenem may be considered if there's suspicion of multi-drug resistant organisms.  3. Route of Administration  Parenteral antibiotics (IV) are typically required initially, especially for severe infections, as they provide higher and more consistent levels of the antibiotic.  Oral antibiotics can be considered for less severe infections or when the patient shows improvement after a few days of IV therapy. Oral options may include clindamycin, trimethoprim-sulfamethoxazole, or fluoroquinolones (e.g., ciprofloxacin).  The transition from IV to oral therapy can be done based on clinical improvement, and typically after 48-72 hours if the infection is controlled and the patient is stable.  4. Duration of Therapy  Bone and joint infections generally require prolonged treatment, typically 4 to 6 weeks, though this can vary based on the severity and clinical response.  In cases of osteomyelitis, a longer course (6-8 weeks) may be needed for complete eradication of the infection.  5. Specific Considerations for Diabetic Patients  Diabetes impairs immune response and increases the risk of infection, making it important to manage blood glucose levels effectively alongside antibiotic treatment to optimize outcomes.  Chronic wounds and poor circulation may require the use of extended courses of antibiotics.  Consideration of diabetic foot care and potential surgical intervention (e.g., debridement) is often part of comprehensive management.  6. Resolution and Recurrence  Adequate surgical intervention (e.g., debridement, drainage, or amputation in severe cases) is often required to prevent recurrence.  The antibiotic regimen should be tailored based on culture results and sensitivity testing to avoid resistance and ensure the most effective treatment.  Regular follow-up and monitoring are crucial to prevent recurrence, particularly given the chronic nature of diabetic foot infections.  Conclusion:  An effective antibiotic regimen for bone or joint infections of the foot in a diabetic patient typically involves IV broad-spectrum antibiotics initially, tailored to culture results. The regimen usually includes coverage for MRSA, gram-negative organisms, and anaerobes. Duration is often extended (4-6 weeks or longer) and must be individualized based on clinical response. Transition to oral antibiotics can occur after initial improvement. Regular monitoring, good glycemic control, and sometimes surgical intervention are key to resolving the infection and preventing recurrence.  **DeepSeek-R1 response:**  In managing bone or joint infections (e.g., osteomyelitis) in diabetic foot infections (DFIs), the antibiotic regimen should be tailored to the patient's clinical status, microbiological findings, and local resistance patterns. Below is a structured summary of evidence-based recommendations:  1. Empiric Antibiotic Selection  Coverage:  Gram-positive pathogens: Include coverage for Staphylococcus aureus (methicillin-sensitive [MSSA] and methicillin-resistant [MRSA]) with agents like vancomycin (IV) or linezolid (oral/IV).  Gram-negative pathogens: Use ceftazidime, cefepime, or fluoroquinolones (e.g., ciprofloxacin) for Pseudomonas aeruginosa in chronic wounds or prior antibiotic exposure.  Anaerobes: Add metronidazole or use a beta-lactam/beta-lactamase inhibitor (e.g., piperacillin-tazobactam).  Local Guidelines: Adjust based on local antibiograms (e.g., MRSA prevalence).  2. Route of Administration  IV vs. Oral:  The OVIVA trial demonstrated non-inferiority of oral antibiotics (e.g., flucloxacillin, clindamycin, or linezolid) compared to IV therapy for bone/joint infections when oral bioavailability is adequate and susceptibility confirmed.  Transition to oral therapy early if clinical improvement, susceptible pathogens, and patient compliance.  3. Duration of Therapy  Standard Duration: 4–6 weeks for osteomyelitis, regardless of route (IV/oral).  Surgical Influence: Shorter courses (e.g., 2 weeks) may suffice if all infected bone is resected, but evidence is limited.  4. Targeted Therapy  MSSA: Switch to beta-lactams (e.g., cephalexin, dicloxacillin) for superior efficacy.  MRSA: Use oral linezolid, doxycycline, or trimethoprim-sulfamethoxazole (if susceptible).  Gram-negatives: Tailor to culture results (e.g., ciprofloxacin for Pseudomonas).  5. Adjunctive Measures  Surgical Debridement: Critical for source control; reduces recurrence and antibiotic duration.  Glycemic Control/Vascular Assessment: Optimize HbA1c and address peripheral arterial disease.  Offloading: Pressure redistribution (e.g., total contact casting) to promote healing.  6. Recurrence Prevention  Monitoring: Follow clinical signs, inflammatory markers (CRP, ESR), and imaging (MRI/X-ray).  Multidisciplinary Care: Podiatry, infectious disease, and endocrinology involvement.  7. Key Guidelines  IDSA 2020 Recommendations:  4–6 weeks of antibiotics for osteomyelitis.  Oral therapy is non-inferior to IV if bioavailable agents are used.  Surgical evaluation for debridement/amputation.  Conclusion  No single regimen is universally superior, but an approach combining early empiric broad-spectrum coverage (adjusted to cultures), oral transition when feasible, 4–6 weeks of therapy, and surgical intervention optimizes outcomes. Recurrence is minimized through comprehensive management addressing infection, ischemia, and metabolic control.  **Grok-3 response:**  Managing bone or joint infections (e.g., osteomyelitis or septic arthritis) in the foot of a person with diabetes is complex due to factors like impaired immune response, poor wound healing, and potential for polymicrobial infections. The choice of antibiotic regimen depends on the causative pathogens, infection severity, and patient-specific factors like renal function or allergies. No single regimen is universally superior, but evidence-based principles guide treatment to optimize resolution and minimize recurrence. Below, I outline key considerations based on available data.  Key Factors Influencing Antibiotic Choice  Pathogens: Diabetic foot infections (DFIs) with bone or joint involvement are often polymicrobial, including:  Gram-positive cocci (e.g., Staphylococcus aureus, including MRSA, and streptococci).  Gram-negative bacilli (e.g., Pseudomonas aeruginosa, Escherichia coli).  Anaerobes (e.g., Bacteroides spp.) in deep or necrotic infections.  Cultures (deep tissue, bone biopsy, or aspirate) are critical to identify pathogens and their antibiotic susceptibilities.  Infection Severity: Bone/joint infections are typically classified as moderate to severe, often requiring prolonged treatment. The Infectious Diseases Society of America (IDSA) and International Working Group on the Diabetic Foot (IWGDF) guidelines categorize DFIs by severity to guide therapy.  Route of Administration:  Parenteral (IV) antibiotics are often used initially for severe infections or when oral bioavailability is inadequate.  Oral antibiotics with high bioavailability (e.g., fluoroquinolones, linezolid) may be suitable for milder cases or step-down therapy after initial IV treatment.  Duration:  Osteomyelitis typically requires 4–6 weeks of therapy, though longer courses (up to 12 weeks) may be needed if surgical debridement is incomplete.  Septic arthritis may require 3–4 weeks, depending on response.  Evidence on Antibiotic Regimens  No randomized controlled trials (RCTs) definitively establish one antibiotic regimen as superior for diabetic foot osteomyelitis or septic arthritis in terms of resolution and recurrence. However, systematic reviews and guidelines (e.g., IDSA 2012, IWGDF 2019, updated through 2023) provide the following insights:  1. Common Antibiotic Regimens  Gram-positive coverage (e.g., MSSA, MRSA, streptococci):  Vancomycin (IV, 15–20 mg/kg every 12 hours, adjusted for renal function): Standard for MRSA until susceptibility is confirmed.  Daptomycin (IV, 6–8 mg/kg daily): Effective for MRSA and vancomycin-resistant enterococci (VRE).  Linezolid (IV/oral, 600 mg every 12 hours): High bioavailability, effective for MRSA, but prolonged use (>2 weeks) risks bone marrow suppression.  Clindamycin (IV/oral, 600–900 mg every 8 hours): Good bone penetration, but resistance in some MRSA strains limits use.  Gram-negative coverage:  Fluoroquinolones (e.g., levofloxacin 750 mg daily, moxifloxacin 400 mg daily, oral/IV): Broad-spectrum, good bone penetration, but resistance in Pseudomonas is rising.  Ceftazidime or cefepime (IV, 1–2 g every 8–12 hours): Effective for Pseudomonas and other Gram-negatives.  Piperacillin-tazobactam (IV, 3.375–4.5 g every 6–8 hours): Broad-spectrum for polymicrobial infections.  Anaerobe coverage:  Metronidazole (IV/oral, 500 mg every 8 hours): Often added for anaerobic involvement.  Clindamycin or amoxicillin-clavulanate (IV/oral, 875/125 mg every 12 hours): Also cover anaerobes.  Combination therapy: Often used in polymicrobial infections (e.g., vancomycin + piperacillin-tazobactam or fluoroquinolone + metronidazole).  2. Route of Administration  Initial therapy: Severe infections or osteomyelitis typically start with IV antibiotics (e.g., vancomycin + ceftazidime) for 1–2 weeks, especially if systemic symptoms (fever, sepsis) are present.  Step-down to oral: High-bioavailability oral agents (e.g., linezolid, fluoroquinolones, rifampin combinations) are effective for completing therapy, especially after surgical debridement. A 2019 meta-analysis (Clinical Infectious Diseases) found no significant difference in outcomes between prolonged IV and oral regimens for osteomyelitis when guided by culture results.  Outpatient parenteral antimicrobial therapy (OPAT): Suitable for patients requiring prolonged IV therapy but stable for outpatient management.  3. Duration of Therapy  Osteomyelitis:  Surgical debridement + antibiotics: 4–6 weeks (IV or oral) is often sufficient if all infected bone is removed.  Non-surgical management: 6–12 weeks, primarily oral if susceptible pathogens.  A 2020 RCT (JAMA) comparing 6 vs. 12 weeks of therapy for osteomyelitis (not specific to diabetic foot) showed no significant difference in remission rates (~70–80%) or recurrence.  Septic arthritis:  3–4 weeks of antibiotics (IV initially, then oral) is typically adequate after joint aspiration or washout.  Recurrence rates are lower with adequate source control (e.g., drainage).  4. Resolution and Recurrence  Resolution rates: Studies report 60–80% resolution with combined surgical and antibiotic therapy, depending on debridement extent and pathogen susceptibility (Clinical Microbiology Reviews, 2019).  Recurrence:  Rates vary (10–30% within 1–2 years), influenced by incomplete debridement, persistent ischemia, or resistant pathogens (e.g., MRSA, Pseudomonas).  Rifampin-based regimens (e.g., rifampin + fluoroquinolone) show promise for staphylococcal osteomyelitis due to biofilm penetration, with some studies reporting lower recurrence (Antimicrobial Agents and Chemotherapy, 2018).  Poor glycemic control (HbA1c >7%) and peripheral vascular disease increase recurrence risk.  Recommendations  Culture-guided therapy: Obtain deep tissue or bone cultures before starting antibiotics to tailor therapy. Avoid empiric therapy beyond 48–72 hours without culture data.  Empiric regimen for severe infection (pending cultures):  Vancomycin (for MRSA) + piperacillin-tazobactam or ceftazidime (for Gram-negatives) + metronidazole (for anaerobes).  Adjust based on culture results and local resistance patterns.  Route and duration:  Start with IV for 1–2 weeks in severe cases, then switch to oral (e.g., levofloxacin + rifampin for staphylococci) if clinically improving.  Total duration: 4–6 weeks for osteomyelitis with debridement; 3–4 weeks for septic arthritis.  Surgical intervention: Debridement or amputation of infected bone/tissue significantly improves outcomes and may shorten antibiotic duration.  Monitoring: Regular follow-up (clinical, imaging, inflammatory markers like CRP/ESR) to assess response and detect recurrence early.  Limitations  No regimen is universally superior due to variability in pathogens, patient comorbidities, and local resistance patterns.  RCTs are limited, and most data come from observational studies or non-diabetic populations.  Resistance (e.g., MRSA, multidrug-resistant Gram-negatives) complicates therapy, emphasizing the need for local antibiograms.  For the most tailored approach, consult an infectious disease specialist and refer to updated IDSA/IWGDF guidelines. If you have specific patient details (e.g., culture results, infection severity), I can refine the recommendation further.  **Claude-3.7 response:**  For diabetic patients with bone or joint infections of the foot (diabetic foot osteomyelitis), the evidence suggests several important considerations for antibiotic therapy, though optimal regimens remain somewhat debated.  Duration of Therapy: The most significant recent evidence comes from studies showing that shorter courses may be as effective as traditionally longer ones. Research suggests that 6 weeks of antibiotic therapy may be sufficient for diabetic foot osteomyelitis, compared to the historically recommended 12+ weeks. This represents a major shift from previous practice patterns.  Route of Administration: Oral antibiotics appear to be as effective as intravenous therapy for many cases, provided the chosen oral agent has good bioavailability and bone penetration. This is particularly relevant for outpatient management and reduces complications associated with long-term IV access.  Specific Agents: The choice should be guided by culture results when possible, but common effective oral options include:  Fluoroquinolones (like ciprofloxacin or levofloxacin) for gram-negative coverage  Clindamycin for anaerobic and some gram-positive coverage  Trimethoprim-sulfamethoxazole for certain gram-positive infections  Newer agents like linezolid for resistant gram-positive organisms  Key Principles:  Empiric therapy should cover common pathogens including Staphylococcus aureus (including MRSA consideration), Streptococcus species, and gram-negative bacteria  Bone biopsy for culture and sensitivity when feasible improves outcomes  Adequate debridement of infected/necrotic tissue is crucial and may be more important than the specific antibiotic chosen  Patient factors like renal function, drug allergies, and ability to take oral medications influence selection  The trend toward shorter courses with oral agents (when appropriate) has improved patient quality of life and reduced healthcare costs while maintaining clinical effectiveness. However, severe infections or those with extensive bone involvement may still require longer treatment courses. |
| 6 | In a person with diabetes and moderate or severe infection of the foot, including osteomyelitis, are there circumstances in which nonsurgical (antibiotic only) treatment is as safe and effective in achieving remission as surgical treatment (combined with antibiotic therapy)？  **ChatGPT-4o response:**  In a person with diabetes and moderate or severe foot infection, including osteomyelitis, surgical treatment combined with antibiotic therapy is generally considered more effective for achieving long-term remission, especially in cases where bone infection (osteomyelitis) is present. However, there are certain circumstances where nonsurgical treatment (antibiotic-only therapy) may be considered as an alternative, but these are limited and depend on the specific situation:  1.Mild osteomyelitis: In some cases of mild osteomyelitis or when there is no significant bone damage, nonsurgical management with appropriate antibiotics might be effective. This is more likely if the infection is localized and there is no extensive involvement of surrounding tissues.  2.Patient factors: For patients who are not good surgical candidates due to other medical conditions (e.g., poor wound healing, significant cardiovascular issues, or advanced age), nonsurgical treatment might be considered if the infection is controlled with antibiotics and there are no signs of systemic infection or widespread tissue necrosis.  3.Bone viability: In cases where bone infection is limited and the bone remains viable (i.e., not significantly necrotic or involved with large abscesses), non-surgical treatment with a prolonged course of antibiotics may achieve remission.  4.Rapid response to antibiotics: Some patients may show a quick response to appropriate antibiotic therapy, especially when the infection is primarily soft tissue and not severe. If clinical signs of infection (e.g., redness, swelling, fever) improve significantly with antibiotics, nonsurgical management might be deemed sufficient.  However, surgical intervention is often preferred because it allows for:  Debridement of necrotic tissue,  Drainage of abscesses,  Removal of infected bone, which significantly reduces the risk of recurrent infection.  In general, surgical treatment combined with antibiotics remains the standard approach for managing moderate to severe infections, particularly when osteomyelitis is involved, to ensure a more thorough resolution and to reduce the risk of complications such as chronic infection or amputation.  **DeepSeek-R1 response:**  In managing diabetic foot infections, including osteomyelitis, the choice between nonsurgical (antibiotic-only) and surgical (combined with antibiotic) treatment depends on several patient-specific and infection-related factors. Here's a structured summary of the circumstances where nonsurgical treatment may be as safe and effective as surgical intervention:  Key Considerations for Nonsurgical Treatment:  1.Infection Characteristics:  Limited Extent: Localized infection without extensive soft tissue necrosis, abscess, or systemic sepsis.  No Significant Bone Destruction: Osteomyelitis involving small, non-weight-bearing bones (e.g., toe phalanges) or minimal bone involvement.  Susceptible Pathogens: Infections caused by organisms with high antibiotic sensitivity, confirmed via culture and susceptibility testing.  2.Patient Factors:  Adequate Vascular Supply: Good perfusion (e.g., absence of significant peripheral artery disease) to ensure antibiotic penetration and wound healing.  Medical Comorbidities: Patients deemed high-risk for surgery due to comorbidities (e.g., severe cardiovascular disease) may benefit from prolonged antibiotics.  Adherence: Willingness and ability to comply with prolonged antibiotic regimens (typically 6–12 weeks) and close follow-up.  3.Guideline Recommendations:  The Infectious Diseases Society of America (IDSA) notes that selected cases of osteomyelitis may be managed medically if there is no residual infected bone or hardware, and if surgical resection is impractical.  Studies (e.g., the VIVID trial) suggest comparable remission rates between medical and surgical groups in carefully selected patients, though recurrence rates may be higher with nonsurgical approaches.  When Surgery is Preferred:  Extensive Necrosis/Abscess: Presence of necrotic tissue, abscesses, or gangrene requiring debridement.  Progressive Infection: Failure of initial antibiotic therapy or clinical deterioration.  Chronic Osteomyelitis with Sequestra: Infected dead bone (sequestrum) that cannot be eradicated with antibiotics alone.  Conclusion:  Nonsurgical treatment with antibiotics alone can be as effective as surgery in achieving remission if the infection is limited, vascular supply is adequate, pathogens are susceptible, and the patient is closely monitored. However, surgical intervention remains the gold standard for complex cases with significant tissue destruction or poor response to antibiotics. Shared decision-making, multidisciplinary care (infectious disease, podiatry, vascular surgery), and individualized assessment are critical to optimizing outcomes.  **Grok-3 response:**  In managing diabetic foot infections (DFIs), including those with osteomyelitis, the choice between nonsurgical (antibiotic-only) and surgical treatment (combined with antibiotics) depends on specific circumstances. Both approaches can be safe and effective in achieving remission in certain scenarios, but the decision hinges on the infection’s severity, extent, patient factors, and clinical context. Below is a detailed analysis based on available evidence and guidelines, tailored to your query.  Key Considerations for Nonsurgical (Antibiotic-Only) Treatment  Nonsurgical treatment with antibiotics alone can be considered safe and effective in achieving remission in some cases of diabetic foot infections, including osteomyelitis, under the following circumstances:  Mild to Moderate Infections Without Extensive Bone Destruction:  For mild to moderate DFIs (e.g., classified by the Infectious Diseases Society of America [IDSA] or International Working Group on the Diabetic Foot [IWGDF] as PEDIS grade 2 or 3), antibiotic therapy alone may suffice if there is no significant necrosis, abscess, or extensive bone involvement.  In cases of osteomyelitis with limited bone involvement (e.g., early-stage, localized infection), prolonged antibiotic therapy (typically 6–12 weeks) can achieve remission without surgery. Studies, such as those reviewed in the 2019 IWGDF guidelines, show that antibiotics alone can be effective for early or limited osteomyelitis, with remission rates comparable to surgical intervention in select cases (approximately 60–80% remission rates).  Absence of Systemic Symptoms or Sepsis:  Patients without systemic signs of infection (e.g., fever, leukocytosis, or hemodynamic instability) are better candidates for nonsurgical management. Systemic symptoms often indicate a need for urgent surgical intervention to control the infection.  Good Soft Tissue and Vascular Status:  Adequate blood supply to the affected area (e.g., palpable pulses or ankle-brachial index >0.9) supports antibiotic delivery to the infection site, increasing the likelihood of success with nonsurgical treatment.  If soft tissue infection is limited and there is no significant necrosis or abscess, antibiotics can effectively penetrate the infected area.  Patient Preference or Surgical Contraindications:  Patients with high surgical risk (e.g., severe comorbidities, poor wound healing potential, or refusal of surgery) may be managed with antibiotics alone if the infection is stable and localized. For example, elderly patients or those with significant cardiovascular disease may benefit from avoiding surgery.  Studies, such as a 2014 retrospective analysis by Tone et al., showed that antibiotic-only treatment achieved remission in 64% of diabetic foot osteomyelitis cases when patients were carefully selected (e.g., no extensive bone destruction or necrosis).  Pathogen-Specific Antibiotic Therapy:  Successful nonsurgical treatment requires precise identification of the causative pathogens (e.g., via bone biopsy or deep tissue culture) and tailored antibiotic therapy. Common pathogens in DFIs include Staphylococcus aureus, streptococci, and gram-negative bacilli. Methicillin-resistant S. aureus (MRSA) may require specific agents like vancomycin or linezolid.  Intravenous antibiotics (e.g., for 2–4 weeks) followed by oral therapy (e.g., for 4–8 weeks) are often used for osteomyelitis, with agents like fluoroquinolones or clindamycin, which have good bone penetration.  When Surgical Treatment (Combined with Antibiotics) Is Preferred  Surgical intervention, typically combined with antibiotics, is often favored in the following scenarios, as it may be safer and more effective:  Severe Infections or Extensive Osteomyelitis:  Severe DFIs (PEDIS grade 4) or osteomyelitis with significant bone destruction, abscess formation, or necrosis often require surgical debridement or resection to remove infected tissue and bone. The 2019 IWGDF guidelines emphasize that surgery is critical when there is extensive necrosis or when antibiotics alone cannot control the infection.  For example, a 2015 study by Aragón-Sánchez et al. found that surgical intervention with antibiotics achieved higher remission rates (85–90%) compared to antibiotics alone (60–70%) in cases of severe osteomyelitis.  Presence of Necrotic Tissue or Abscess:  Necrotic tissue or abscesses impair antibiotic penetration, reducing the effectiveness of nonsurgical treatment. Surgical debridement removes these barriers, enhancing antibiotic efficacy and promoting healing.  Progressive Infection or Systemic Involvement:  Patients with systemic symptoms (e.g., sepsis, high fever, or elevated inflammatory markers like C-reactive protein) or progressive infection despite antibiotics typically require surgery to achieve source control.  Poor Response to Initial Antibiotic Therapy:  If clinical or radiographic evidence (e.g., persistent bone destruction on MRI or X-ray) shows no improvement after 2–4 weeks of appropriate antibiotics, surgical intervention is often necessary to prevent amputation or chronic infection.  Anatomic or Functional Considerations:  Surgery may be preferred to preserve foot function or prevent complications like gangrene. For example, resection of infected bone in the forefoot may prevent the need for major amputation.  Comparing Safety and Effectiveness  Safety:  Antibiotic-only treatment: Generally safer in terms of avoiding surgical risks (e.g., anesthesia complications, wound healing issues, or postoperative infections). However, prolonged antibiotic use carries risks of adverse effects (e.g., Clostridioides difficile infection, hepatotoxicity, or antibiotic resistance).  Surgical treatment: Carries perioperative risks, particularly in patients with diabetes who may have poor wound healing or comorbidities. However, surgery reduces the risk of uncontrolled infection leading to sepsis or amputation.  Effectiveness:  Antibiotic-only treatment: Effective in carefully selected cases (e.g., early osteomyelitis, good vascular supply, no necrosis), with remission rates of 60–80% in studies like Game et al. (2012). However, recurrence rates may be higher (20–30%) compared to surgical approaches.  Surgical treatment: Generally more effective for severe or extensive infections, with remission rates of 85–90% in studies. Surgery addresses underlying necrosis and reduces bacterial load, lowering recurrence risk (10–20%).  Clinical Decision-Making  The decision to pursue nonsurgical versus surgical treatment should be guided by:  Infection severity: Use classification systems like IDSA/IWGDF (PEDIS) to assess severity.  Imaging: MRI, CT, or X-ray to evaluate bone involvement and necrosis.  Vascular assessment: Ensure adequate perfusion (e.g., Doppler ultrasound or ankle-brachial index).  Microbiology: Obtain cultures to guide antibiotic therapy.  Multidisciplinary input: Involve infectious disease specialists, podiatrists, and vascular surgeons.  Patient factors: Consider comorbidities, surgical risk, and patient preferences.  Practical Example  A patient with a moderate DFI and early osteomyelitis (e.g., localized to a single metatarsal, no abscess, good vascular supply) may achieve remission with 6–12 weeks of targeted antibiotics (e.g., oral levofloxacin and rifampin for S. aureus). However, if imaging shows extensive bone destruction or the patient develops an abscess, surgical debridement followed by 4–6 weeks of antibiotics would likely be more effective and reduce recurrence risk.  Conclusion  Nonsurgical (antibiotic-only) treatment can be as safe and effective as surgical treatment for diabetic foot infections, including osteomyelitis, in cases of mild to moderate infection, limited bone involvement, good vascular supply, and absence of necrosis or systemic symptoms. However, surgical treatment is generally preferred for severe infections, extensive osteomyelitis, or when necrosis or abscesses are present, as it achieves higher remission rates and lower recurrence risks. Individualized assessment, guided by clinical, imaging, and microbiological data, is critical to optimize outcomes.  If you need further details or a specific case analysis, please provide additional information (e.g., infection severity, imaging findings, or patient comorbidities).  **Claude-3.7 response:**  For diabetic foot infections with osteomyelitis, the choice between surgical and nonsurgical treatment depends on several clinical factors, and there are indeed circumstances where antibiotic-only treatment can be as effective as surgical intervention.  Circumstances favoring nonsurgical treatment:  Patient factors:  Patients who are poor surgical candidates due to severe comorbidities, advanced age, or high perioperative risk  Limited life expectancy where aggressive intervention may not be appropriate  Patient preference after informed discussion of risks and benefits  Infection characteristics:  Early-stage osteomyelitis without extensive bone destruction  Infections caused by organisms highly sensitive to oral or intravenous antibiotics  Absence of large sequestra (dead bone fragments) or extensive cortical destruction  Good vascular supply to the affected area  Clinical presentation:  Absence of systemic sepsis or severe systemic illness  No large abscesses or extensive soft tissue necrosis  Adequate perfusion and potential for healing  Evidence supporting nonsurgical treatment:  Recent studies have shown that in carefully selected patients, prolonged antibiotic therapy (typically 6-12 weeks) can achieve remission rates comparable to surgical debridement plus antibiotics. Success rates for antibiotic-only treatment in appropriate candidates range from 60-80% in various studies.  Key considerations:  The decision requires careful evaluation of bone destruction extent (via imaging), vascular status, infection severity, and overall patient condition. Close monitoring is essential with nonsurgical treatment, as failure may necessitate eventual surgical intervention.  Monitoring requirements:  Serial inflammatory markers (ESR, CRP)  Clinical assessment of wound healing  Imaging to assess bone changes  Vascular assessment  The approach should be individualized, with shared decision-making between patient and healthcare team considering all relevant factors. |
| 7 | In a person with diabetes and a foot infection, does the addition of any specific adjunctive or topical antibiotic treatment to systemic antibiotic therapy and surgery improve the outcome of infection？  **ChatGPT-4o response:**  In patients with diabetes and foot infections, the addition of adjunctive or topical antibiotic treatments to systemic antibiotic therapy and surgical intervention can sometimes be beneficial, but it depends on the specifics of the infection and the overall treatment plan. Here are key points to consider:  Systemic Antibiotics: These are essential for treating the infection as they reach deeper tissues and can address the full extent of the infection, especially in diabetic patients who are more susceptible to serious infections due to compromised immune function.  Topical Antibiotics: For superficial or localized infections, topical antibiotics like mupirocin or silver sulfadiazine can be useful. However, they typically are less effective in deeper infections, as they do not penetrate the tissue as effectively as systemic antibiotics.  Adjunctive Antibiotics: In some cases, adjunctive therapies (like the use of oral or intravenous antibiotics targeting specific bacteria, such as MRSA or Pseudomonas) may improve outcomes. This depends on the type of bacteria identified and the severity of the infection. For example, adding antibiotics that target specific organisms not covered by the initial regimen can help prevent resistance or improve healing.  Wound Care and Debridement: Surgical intervention, such as debridement (removal of necrotic tissue), is often a crucial component in treating diabetic foot infections. This helps reduce bacterial load and promotes better antibiotic penetration.  Increased Risk of Resistance: Overuse of topical antibiotics can lead to local resistance, especially if used excessively. Hence, they should generally complement, rather than replace, systemic therapy.  Outcome Improvement: Evidence suggests that while topical antibiotics may not drastically change the outcomes of deep or severe infections, they can aid in the healing of surface-level wounds or in preventing reinfection in combination with proper systemic therapy and surgical treatment.  In summary, the addition of topical or adjunctive antibiotic therapy can be beneficial in certain situations, particularly for superficial infections or to prevent recurrence, but systemic antibiotics and surgical intervention remain the cornerstone of treatment in diabetic foot infections. Always tailored to the specific infection and guided by microbial culture and sensitivity testing.  **DeepSeek-R1 response:**  In managing diabetic foot infections, the addition of specific adjunctive or topical antibiotics to systemic antibiotic therapy and surgery does not consistently improve clinical outcomes. Here's a synthesized analysis:  Key Considerations:  1.Guideline Recommendations:  IDSA (2020) and IWGDF Guidelines: Do not recommend topical antimicrobials for infected diabetic foot ulcers due to insufficient evidence of benefit. Emphasis remains on systemic antibiotics tailored to infection severity and pathogens, alongside surgical debridement.  2.Topical Antibiotics:  Limited Penetration: Effective delivery to deep tissues is challenging, especially in necrotic or ischemic areas. Their utility may be restricted to superficial infections after debridement.  Mixed Evidence: Studies on agents like cadexomer iodine or silver show conflicting results, often demonstrating reduced bacterial load without significant improvement in healing or amputation rates.  Risks: Potential for contact dermatitis, antibiotic resistance, and minimal added benefit in severe infections already treated systemically.  3.Adjunctive Therapies:  Biofilm Disruption: While biofilm-targeting agents (e.g., certain dressings) are theorized to enhance systemic antibiotic efficacy, robust clinical evidence is lacking.  Non-Antibiotic Adjuncts: Hyperbaric oxygen, growth factors, or maggot therapy focus on tissue repair rather than direct antimicrobial effects, with variable evidence supporting their use.  4.Clinical Outcomes:  Severity-Dependent: Topical agents may offer marginal benefits in mild infections but are unlikely to impact moderate-severe cases managed with systemic therapy and surgery.  No Significant Impact: Meta-analyses and RCTs generally fail to show improved resolution of infection, reduced amputations, or mortality with adjunctive topicals.  Conclusion:  Current evidence does not support routine use of topical or adjunctive antibiotics in diabetic foot infections treated with systemic antibiotics and surgery. Guidelines prioritize systemic therapy and surgical intervention, reserving topical agents for select superficial cases. Further research is needed to identify niche scenarios where adjunctive treatments might add value.  **Grok-3 response:**  In people with diabetes and foot infections, the addition of specific adjunctive or topical antibiotic treatments to systemic antibiotic therapy and surgery may improve outcomes in certain cases, but the evidence is mixed and depends on the context.  Key Points from Available Evidence:  Systemic Antibiotics as Primary Treatment: Systemic antibiotics are the cornerstone of treatment for diabetic foot infections (DFIs), alongside surgical interventions like debridement or drainage when needed. The choice of antibiotics depends on the infection’s severity, likely pathogens, and local resistance patterns.  Topical Antibiotics:  Limited Evidence for Benefit: Topical antibiotics (e.g., gentamicin, silver-based agents) may be considered for mild infections or as adjuncts in moderate-to-severe cases, but robust evidence showing significant improvement in outcomes (e.g., infection resolution, amputation prevention) is limited.  Specific Agents: Studies suggest topical agents like pexiganan (a magainin peptide) or gentamicin-collagen sponges may reduce bacterial load in some cases, but they don’t consistently improve clinical outcomes compared to systemic therapy alone.  Silver-Based Products: Silver dressings or creams are commonly used for their antimicrobial properties, but high-quality randomized controlled trials (RCTs) show no clear superiority over standard care in healing or infection resolution.  Adjunctive Therapies:  Hyperbaric Oxygen Therapy (HBOT): Some studies indicate HBOT may reduce amputation rates in severe DFIs by improving tissue oxygenation and wound healing, but results are inconsistent, and it’s not universally recommended.  Granulocyte Colony-Stimulating Factor (G-CSF): Limited evidence suggests G-CSF might reduce the duration of infection or hospitalization in some patients, but it doesn’t significantly lower amputation rates.  Negative Pressure Wound Therapy (NPWT): NPWT can promote wound healing and reduce bacterial load when used post-surgery, but its impact on infection resolution is not well-established compared to standard care.  Challenges and Considerations:  Infection Severity: Mild infections may not require topical or adjunctive therapies, while severe infections (e.g., osteomyelitis) often need prolonged systemic antibiotics and surgery, with topical treatments playing a minor role.  Antibiotic Resistance: Overuse of topical antibiotics can contribute to resistance, limiting their utility.  Individual Factors: Patient-specific factors like vascular status, glycemic control, and comorbidities significantly influence outcomes, often more than the choice of adjunctive therapy.  Guidelines and Recommendations:  The Infectious Diseases Society of America (IDSA) and International Working Group on the Diabetic Foot (IWGDF) emphasize systemic antibiotics and surgical intervention as primary treatments. Topical antibiotics are not routinely recommended but may be considered for specific cases (e.g., mild infections with poor systemic drug penetration).  Adjunctive therapies like HBOT or NPWT should be reserved for complex cases (e.g., non-healing wounds or severe ischemia) and guided by specialist input.  Conclusion: While topical antibiotics and adjunctive therapies may offer some benefits in specific scenarios, there’s no strong evidence that they consistently improve infection outcomes beyond systemic antibiotics and surgery. Treatment should be tailored to infection severity, patient factors, and local guidelines, with a focus on optimizing systemic therapy and surgical management.  If you’d like me to search for more recent studies or specific trials on this topic, let me know!  **Claude-3.7 response:**  In diabetic foot infections, the evidence for adding specific adjunctive or topical antibiotic treatments to standard systemic therapy and surgery is mixed, with limited high-quality data supporting routine use.  Current Evidence:  Topical Antibiotics:  Most clinical guidelines do not routinely recommend topical antibiotics as adjunctive therapy for diabetic foot infections  Limited penetration into infected tissue, especially in the presence of biofilms and necrotic tissue  Risk of developing antimicrobial resistance  May be considered in specific cases with exposed bone or after surgical debridement  Adjunctive Therapies with Some Evidence:  Hyperbaric Oxygen Therapy (HBOT):  Some studies suggest benefit as adjunctive treatment for severe diabetic foot infections  May improve tissue oxygenation and enhance antibiotic efficacy  Evidence remains controversial with mixed results in randomized trials  Negative Pressure Wound Therapy:  Can be beneficial after surgical debridement  Helps with wound healing but doesn't directly treat infection  Antiseptic Solutions:  Dilute povidone-iodine or chlorhexidine may have some role in wound care  Should not replace systemic antibiotics  Standard of Care Remains:  Appropriate systemic antibiotic therapy based on culture and sensitivity  Aggressive surgical debridement when indicated  Glycemic control  Pressure offloading  Wound care  Bottom Line: The cornerstone of treatment remains systemic antibiotics appropriate for the cultured organisms, combined with surgical intervention when necessary. Most adjunctive topical treatments have not shown consistent benefit in well-designed studies, and routine use is not recommended by major clinical guidelines including those from the Infectious Diseases Society of America.  Individual cases may warrant consideration of adjunctive therapies, but this should be based on specific clinical circumstances and ideally in consultation with infectious disease and wound care specialists. |
